# Supramolecular ICT Modulation in π‑Extended 2D-Azeno Nanosheets for Reversible Fluorescence Sensing of Spermine at the Sub-10 nM Level

**DOI:** 10.1021/acs.analchem.6c02145

**Published:** 2026-07-31

**Authors:** Priyanka Rana, Deepak Dabur, Hui-Fen Wu

**Affiliations:** † Department of Chemistry, 34874 National Sun Yat-Sen University, Kaohsiung, 70, Lien-Hai Road, Kaohsiung 80424, Taiwan; ‡ School of Pharmacy, College of Pharmacy, Kaohsiung Medical University, Kaohsiung 807, Taiwan; § Institute of Medical Science and Technology, National Sun Yat-Sen University, Kaohsiung 80424, Taiwan; ∥ School of Medicine, College of Medicine, National Sun Yat-Sen University, Kaohsiung 80424, Taiwan; ⊥ Institute of Precision Medicine, National Sun Yat-Sen University, Kaohsiung 80424, Taiwan; # Institute of Biopharmaceutical Science, National Sun Yat-Sen University, Kaohsiung 80424, Taiwan

## Abstract

Biogenic amine surveillance is central to food safety and clinical diagnostics, yet real-time, selective detection of spermine (SPM) remains challenging due to molecular similarity among amines and probe instability in complex matrices. Here, we report a reusable, wavelength-shift-responsive fluorescent probe based on two-dimensional azene nanosheets (2D-Azeno NSs), synthesized through a Schiff condensation route followed by ultrasonic exfoliation. The robust, π-extended framework exhibits intense green photoluminescence (λ_em_ = 501 nm) and achieves an ultralow detection limit of 4.35 nM via a unique ratiometric fluorescence response accompanied by a distinct hypsochromic shift. Mechanistic interrogation, supported by DFT calculations, reveals that selective sensing arises not from conventional Inner Filter Effects but from site-specific, cooperative hydrogen bonding between the polyamine functional groups of SPM and the N–H/C=N sites of the 2D-Azeno scaffold. This supramolecular recognition selectively perturbs the Intramolecular Charge Transfer (ICT) pathway, leading to a measurable widening of the frontier orbital energy gap creating a Hydrogen-bond induced charge transfer modulation (HICTM) sensor. Furthermore, this sensing pathway is inherently reversible; acid fuming protonates the amine sites, disrupting the H-bond network and fully regenerating the initial emission state over nine operational cycles. This work establishes a paradigm for supramolecular control over a solid-state electronic structure in rigid 2D organic materials, offering a generalizable design principle for developing robust, regenerable, and high-fidelity optical sensors applicable to environmental monitoring and precision biomedicine.

## Graphical abstract

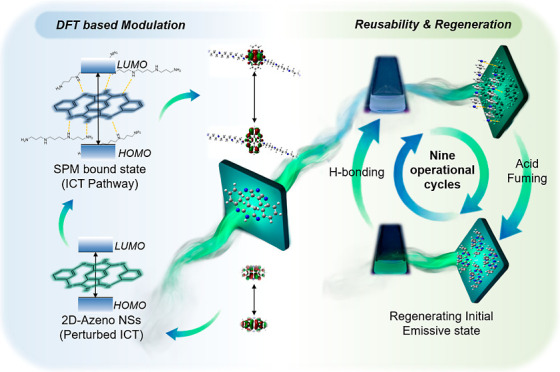

## Introduction

Amines are ubiquitous biomolecules that perform essential structural and regulatory functions in living systems, serving as key components of amino acids, neurotransmitters, and other biologically active compounds. However, when their concentrations exceed normal physiological limits, certain amines may indicate environmental pollution, industrial discharge, or food degradation.^1,2^ With the increasing globalization of food distribution, maintaining freshness and safety during storage and transportation has become a critical challenge, particularly for perishable commodities.^3^ During cellular and microbial spoilage, amino acids undergo enzymatic or thermal decarboxylation, leading to the formation of biogenic amines (BAs) such as histamine, cadaverine, putrescine, tyramine, spermidine, spermine, and ethanolamine. The accumulation of these compounds is widely recognized as a reliable biochemical marker for assessing food quality and hygienic condition.^3–5^ Moreover, excessive intake of BAs can induce various toxicological effects, including hypertension, headaches, respiratory distress, and gastrointestinal discomfort.^6,7^ In particular, BAs produced through microbial metabolism in decomposed food can provoke severe allergic or toxic reactions manifesting as hypotension, skin irritation, headaches, and, in extreme cases, even fatality.^7^ Among these, spermine (SPM), an aliphatic biogenic amine, is commonly detected in food matrices such as pork, mushrooms, and corn. SPM can readily react with nitrite to generate nitrosamines, a class of potent carcinogenic compounds that pose significant risks to food safety.^8,9^ Elevated concentrations of SPM in body fluids have also been linked to adverse physiological outcomes, including nausea, headaches, vomiting, cardiac palpitations, and malignant tumors.^10,11^ Consequently, developing a highly selective, accurate, and sensitive analytical strategy for SPM detection is of paramount importance to safeguard consumer health and ensure food quality throughout the supply chain.

The reliable detection and discrimination of volatile amines that threaten environmental and food safety standards remain a formidable challenge for modern analytical chemistry. Among the myriad of sensing platforms, impedimetric transducers have attracted considerable attention because of their subnanomolar detection limits; however, their analytical fidelity is often compromised by nonspecific ionic or molecular interferences that mask the target signal and diminish selectivity.^12^ Additionally, traditional techniques using immunoassays, ion exchange liquid chromatograph, high-performance liquid chromatography (HPLC), and other commercial approaches offer high sensitivity and excellent separation but are hampered by difficult and time-consuming laborious processing.^11^ In contrast, fluorescence-based sensors have emerged as a powerful alternative owing to their contact-free operation, ultrafast response times, and exquisite sensitivity that can reach the picomolar regime.^13–15^ Consequently, a plethora of emissive probes have been engineered for real-time monitoring of food freshness and biogenic-amine accumulation, delivering encouraging performance metrics.^16,17^ Yet, the majority of these fluorescent systems suffer from intrinsic drawbacks that limit their practical applicability like photobleaching of the chromophore under continuous excitation, which shortens the usable lifetime of the probe,^18^ light scattering and matrix-induced quenching in heterogeneous food matrices, which deteriorate signal-to-noise ratios,^19,20^ and poor chemoselectivity towards structurally similar amines such as spermine (SPM),^15–17^ leading to false-positive readouts. Moreover, most reported designs rely on extrinsic organic dyes or metal-complex emitters to generate fluorescence, and the quenching event is typically irreversible, precluding sensor regeneration and inflating assay cost.^15,21–23^ These limitations underscore the urgent need for intrinsically robust, dye-free, and reversible fluorescent materials that can maintain high quantum yields, resist photodegradation, and provide selective, recyclable detection of target amines in complex environments.

In this work, we report a fluorescence-based probe that transcends the intrinsic shortcomings of conventional amine sensors by delivering exceptionally high selectivity, reversible quenching towards spermine (SPM), and robust reusability. The probe’s fluorescence can be switched off and on through successive sensing–desorption cycles without any significant deterioration in performance, ensuring long-term operational stability and cost-effectiveness in complex food matrices. The sensing mechanism is rationalized by density-functional-theory (DFT) calculations that reveal a dominant hydrogen-bonding interaction between the primary amine of SPM and the polar amine sites of the probe, while the molecular architecture is anchored on a π-conjugated two-dimensional azene (2D-Azeno) scaffold generated via condensation of *m*-phenylenediamine with pyromellitic diimide. This scaffold furnishes a dense array of carbonyl and amine functionalities that act as electron-donating and polar motifs, fostering C=N linkages and extensive π–π stacking that drive the self-assembly of a layered supramolecular framework. Ultrasonication exploits these intermolecular forces to exfoliate the material into atomically thin nanosheets, which are further stabilized in-plane by intermolecular nitrile linkages and out-of-plane by π–π interactions, yielding a highly ordered 2D architecture that underpins the probe’s selective, reversible, and wavelength-tunable fluorescence response.

The resulting 2D-Azeno nanosheets exhibit highly stable, deep-green fluorescence that remains consistent across a broad range of temperature conditions. This exceptional photostability and environmental robustness make 2D-Azeno particularly well-suited for biogenic amine detection, as it maintains a strong emission intensity and structural integrity even under the variable and often harsh conditions encountered in real food matrices. These features, combined with its hydrogen-bonding-driven selectivity and reversible optical response, position the 2D-Azeno HICTM probe as a next-generation sensing material for the precise and practical detection of SPM and related biogenic amines.

## Materials and Methods

### Chemicals and Reagents

*m*-Phenylenediamine and pyromellitic diimide were purchased from Tokyo Chemical Industry (TCI) and Thermo Fischer Scientific, respectively. The solvents used during the solvent study (ethanol, dimethyl sulfoxide, hexane, acetonitrile, tetrahydrofuran, toluene, methanol, acetone, and water) were of ACS reagent grade and were used as is without any further purification. All the biogenic amines, metal salts, and interfering analytes utilized during the applicability of the sensor were purchased from Sigma-Aldrich, USA.

### Instrumentation

An EVOLUTION 201 (Thermo SCIENTIFIC, USA) UV–vis absorption spectrophotometer and Hitachi Fluorescence Spectrophotometer F-2700 (Hitachi, Japan) were used to study the optical properties (absorption and fluorescence) of the as-synthesized material. The morphological characterization of the material was done with the help of a JEOL (Japan) JEM2100 transmission electron microscope (TEM) and a field emission scanning electron microscope (6700). The structural characterization of the material was done by using X-ray diffraction (XRD, Bruker D8, Advance, Philips, Netherlands) for crystallinity, an Auger electron spectrometer (JEOL, Japan) for XPS, and a JEOL NMR 400 MHz for nuclear magnetic resonance (NMR) spectra. Additionally, an ELSZ-2000 (Otsuka Electronic, Japan) dynamic light scattering (DLS) instrument was used to analyze the sizes of the prepared material.

### Synthesis

As illustrated in Figure 1a, 2D-Azeno was synthesized using *m*-phenylenediamine and pyromellitic diimide. The synthesis has been done by following the traditional route for the synthesis of Schiff’s base by using ethanol and acetic acid (1:1) as a solvent medium. *m*-phenylenediamine (2 mol.) and pyromellitic diimide (1 mol.) were simply added to the reaction medium and stirred at room temperature for 30 min. After 30 min, the temperature was increased up to 120 °C, and the reaction mixture was stirred for 24 h to obtain the bulk 2D-Azeno. The reaction mixture was further subjected to Probe Ultrasonication for 40 min (3s ON, 2s OFF) in order to undergo exfoliation and conversion into 2D NSs.

**Figure 1:**
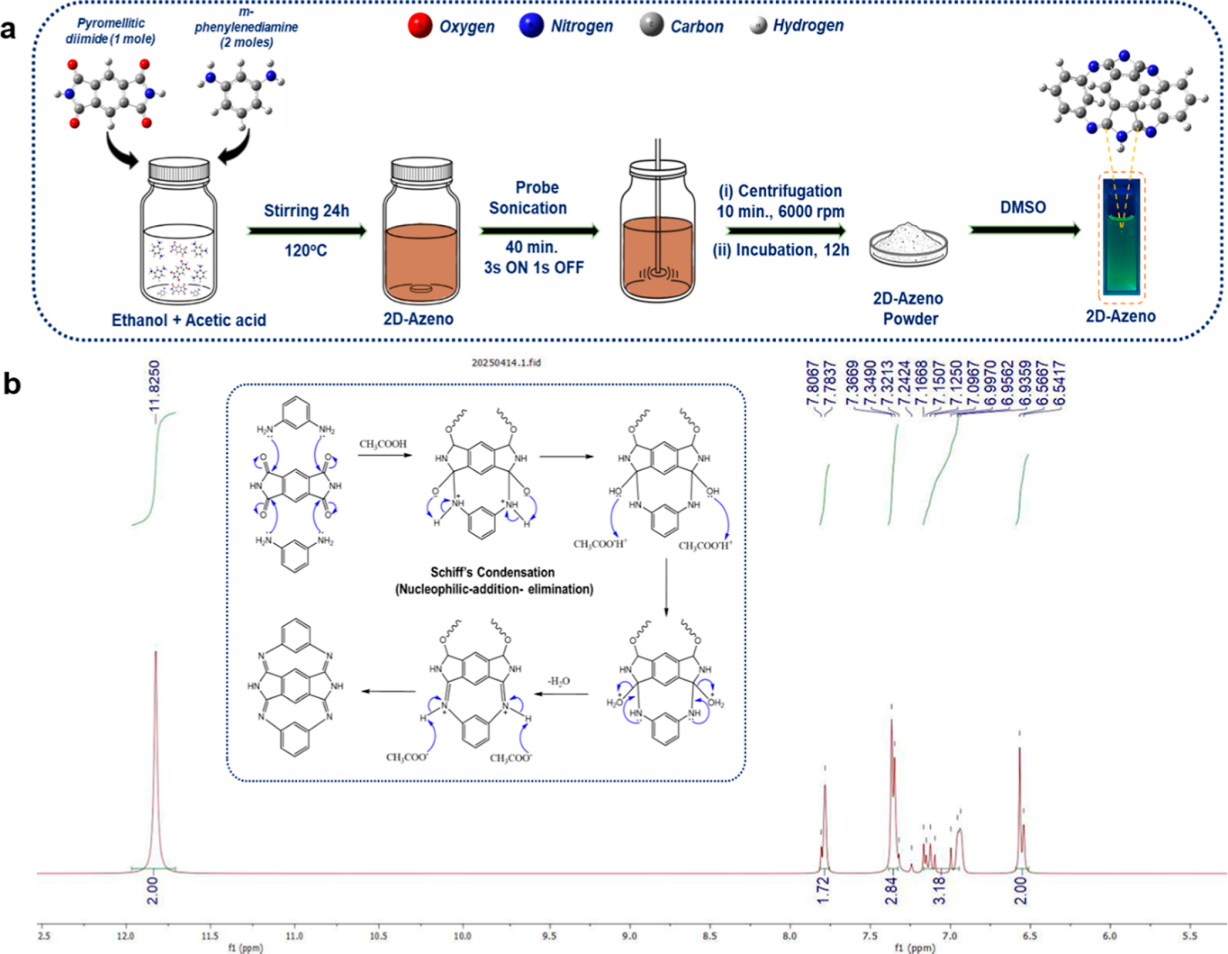
(a) Schematic illustration for the synthesis of 2D-Azeno. (b) Nuclear magnetic resonance (NMR) spectra for 2D-Azeno, and the inset shows the stepwise reaction mechanism for the formation of 2D-Azeno via nucleophilic addition–elimination.

Furthermore, the obtained solution was centrifuged, and the powder was separated, washed with hexane, and dried to obtain 2D-Azeno NSs. The 2D-Azeno powder was dispersed (1 mg/mL) in different solvents to study the effect of solvent medium on optical properties, and as a result, DMSO was chosen as the suitable solvent for further studies. The reaction mechanism for the Schiff’s condensation as demonstrated by the Figure 1b inset follows a two-step nucleophilic addition elimination pathway starting with the formation of a carbinolamine intermediate due to the nucleophilic attack of *m*-phenylenediamine (primary amine) on the carbonyl carbon of pyromellitic diimide (ketone). In the presence of acetic acid (an acid catalyst), the dehydration of intermediate produces a Schiff base (2D-Azeno) containing azomethine­(C=N) bonds.

### Spermine (SPM) Detection Method

A 5 mM stock solution of SPM was prepared and employed during the detection process. A total volume of 1 mL was prepared for the detection of SPM by mixing an increasing concentration of SPM (5 nM–450 μM) with 2D-Azeno NS solution, and the emission spectra were recorded at an excitation wavelength of 400 nm. Each experiment was conducted in at least three replicates to test the designed sensor’s accuracy.

### Selectivity and Interference

Sensor selectivity toward biogenic amines was tested by analyzing structurally and functionally comparable amines. The emission spectra at 400 nm excitation wavelength was analyzed for selectivity studies by mixing 200 μM of the biogenic amines, namely, ammonia, methyl amine, propyl amine, diphenyl amine, triethyl amine, *N*,*N*-diethyl amine, putrescine, cadaverine, spermidine and SPM with 2D-Azeno solution in DMSO. The possible interfering analytes present in the biological systems and the meat samples including metal ions have also been analyzed by mixing 200 μM of SPM with 2D-Azeno solution in the presence of 5 mM of the interfering substances when excited at 400 nm.

### Real Sample Preparation for Qualitative and Quantitative Analyses

The feasibility of the sensor was further extended to real biological samples of human serum and urine as well as the meat samples of chicken and shrimp. The biological samples (human serum and urine) were pretreated by centrifugation followed by filtration, dilution, and then spiked with known concentrations of SPM and were mixed with 2D-Azeno solution in DMSO to prepare a final volume of 1 mL. To analyze the meat samples for freshness, the fresh chicken and shrimp samples were bought from the local market, stored at two different temperatures (25 °C and −10 °C) in the presence of the as-prepared 2D-Azeno NS sensor, and were analyzed over a course of 18 h under a UV lamp to ensure the applicability of the sensor if the SPM is present in the gaseous phase.

### DFT and TD-DFT Calculations

The DFT and TD-DFT calculations were done using the Gaussian 16W with suitable functionals and basis sets. As shown in Table S1, all the calculations throughout the studies were done using the B3LYP functional and 6-31­(d,p) basis set along with implicit solvation effects included with the help of the Self-Consistent Reaction Field (SCRF) model. All the molecular orbitals and the optimized geometries were visualized using GaussView 6.0. For accurate UV–vis absorption spectra, empirical dispersion correction basis set em = gd3 was applied in order to include both hydrogen bonding and π–π interactions for 2D-Azeno.

## Results and Discussion

### Characterization and Structural Properties

The synthetic route and the corresponding reaction mechanism for the as synthesized 2D-Azeno NS has been illustrated in the Figure 1a,b inset, respectively. The structure of 2D-Azeno is confirmed with ^1^H NMR (400 MHz, DMSO-*d*_6_): 11.8 (2H, s), 7.7 (d, 2H), 7.34 (dd, 3H), 7.1 (m, 3H), and 6.5 (d, 2H) as highlighted in Figure 1b. The morphological characterization of the 2D-Azeno NS has been given in Figure 2. Figure S1a,b displays the transmission electron microscopy (TEM) image for the bulk-Azeno NS obtained after stirring (scale bar = 1 μm) and selected-area electron diffraction (SAED) pattern, confirming its polycrystalline nature, respectively. The high-angle annular dark field scanning transmission electron microscopy (HAADF-STEM) images of the exfoliated 2D-Azeno NS prepared via probe sonicating the bulk-Azeno has been provided in Figure 2a, indicating the successful formation of the 2D material after exfoliating the bulk material. Figure 2b shows the SAED pattern of 2D-Azeno having a single-crystal nature along with the sheet-like morphology (scale bar = 200 nm). The nanosheet morphology of 2D-Azeno has been further evidenced by Scanning electron Microscopy (SEM) images as displayed in Figure 2c, showing a layered structure which has been further confirmed by Atomic Force Microscopy (AFM) images and height profile having an average thickness of 42.91 nm (Figure S2a). The elemental distribution (Figures 2d and S2b) for 2D-Azeno NSs shows a uniform distribution of nitrogen (green color) as well as carbon (pink color). The dynamic light scattering (DLS) spectra for 2D-Azeno NSs are provided in Figure 2e, suggesting that the majority of the nanosheets are in the range of 400 nm. In order to investigate the crystalline nature of the 2D-Azeno NSs, X-ray diffraction (XRD) spectra have been analyzed as depicted by Figure 2f, and the major peaks were found to be at 2 theta values 17.27°, 18.38°, 18.89°, 21.01°, 29.49°, 33.83°, and 42.22° with monoclinic crystal lattice referenced by JCPDS No. 98-024-8238. The successful bond formation between the ketonic moiety of pyromellitic diimide and the amine group of *m*-phenylenediamine has been further backed up by X-ray Photoelectron Spectroscopy (XPS) (Figure 2g,h) and Fourier transform infrared spectra (FTIR) studies (Figure 2i). The high-resolution XPS spectra shown in Figure 2g for C 1s showed three distinct peaks at binding energy values at 284.8 eV, 285.06, and 288.26 eV owing to the presence of C=C/C–C, C–N, and N–C=N bonds, respectively, in the structure of 2D-Azeno NSs.^24,25^ Figure 2h shows the high-resolution XPS spectra for N 1s showing two peaks at binding energy values 399.67 and 400.26 eV corresponding to the C–NH and N=C bonds, respectively.^26,27^ The FTIR analysis was also performed to confirm the formation of 2D-Azeno as displayed in Figure 2i; the two N–H peaks present in the precursor *m*-phenylenediamine at higher frequency due to asymmetric stretching and at lower frequency due to symmetric N–H stretching have disappeared after the formation of 2D-Azeno. Additionally, the typical peak for C=O at around 1768 cm^–1^ in the starting material pyromellitic diimide has disappeared, and a new peak at 1715 cm^–1^ and 1557 cm^–1^ owing to the formation of C=N has appeared in 2D-Azeno. All other peaks for the precursors remained almost intact after the reaction, with slight shifts due to the changes in bond environments. Also, a broad peak between 2900 and 3300 cm^–1^ was also observed in the FTIR spectra for 2D-Azeno, which can be attributed to the residual solvent traces after the reaction.

**Figure 2:**
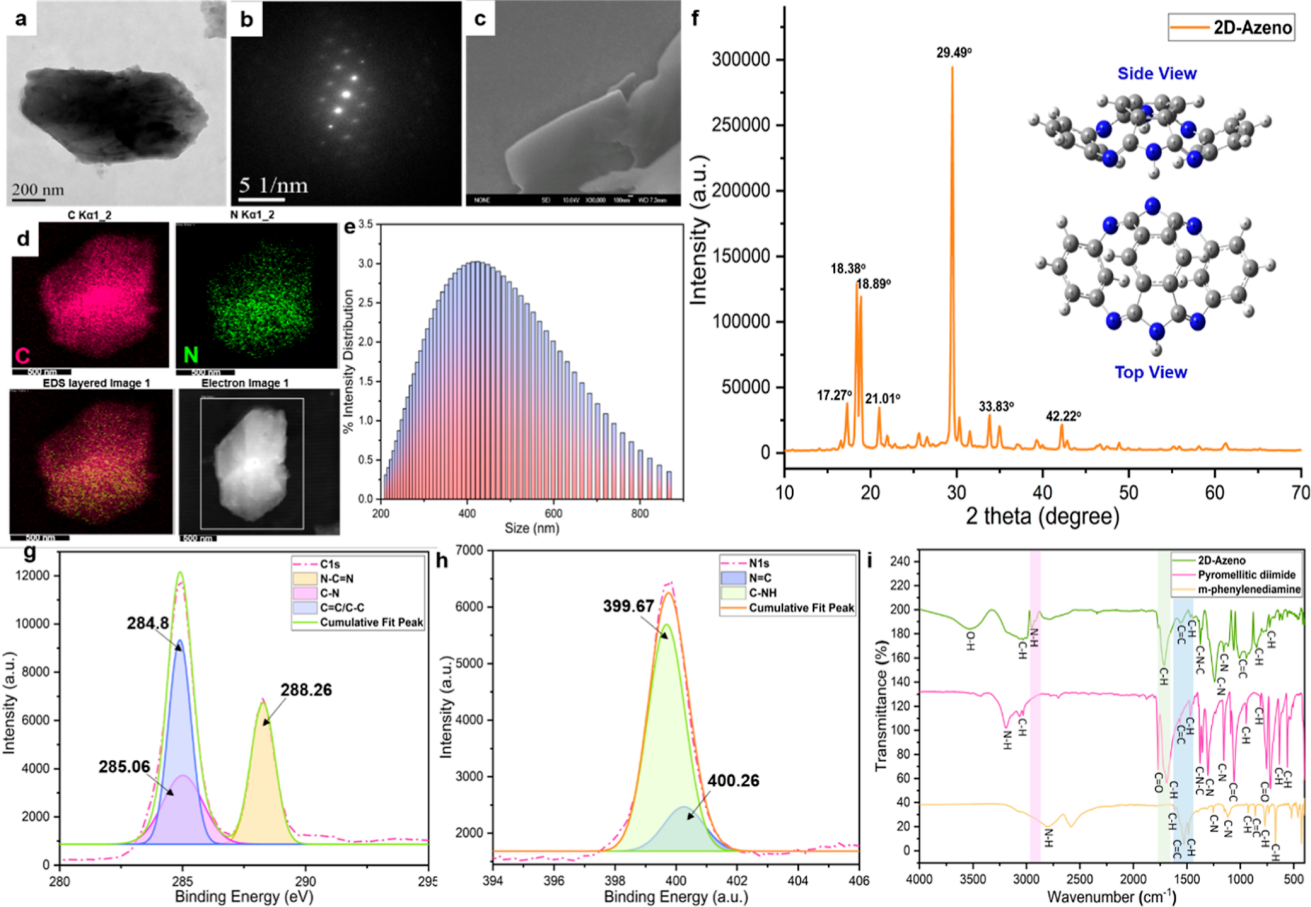
Structural properties of 2D-Azeno NSs. (a)­Transmission electron microscope (TEM) image for 2D-Azeno (200 nm scale); (b) SAED pattern; (c) scanning electron microscopy (SEM) images of 2D-Azeno; (d) HAADF-STEM image, elemental distribution for 2D-Azeno NSs (pink represents carbon and green represents nitrogen), and HAADF-STEM electron image for 2D-Azeno NSs; and (e) DLS spectra of 2D-Azeno NSs. (f) XRD data for 2D-Azeno along with the structure of 2D-Azeno NSs, (g) C 1s XPS spectra, (h) N 1s XPS spectra, and (i) FTIR spectra for control and 2D-Azeno NSs.

### Optical Properties

The optical properties of the as-synthesized material including UV–vis spectra and Photoluminescence (PL) spectra have been mentioned in Figure 3. Figure 3a presents the UV absorbance of 2D-Azeno NSs showing a significant difference and shift in the absorption peaks when compared to the precursors pyromellitic diimide and *m*-phenylenediamine. The 2D-Azeno NSs showed two prominent absorbance peaks at around 263 and 307 nm, which has been further evidenced by DFT studies (Table S2 in the Supporting Information). The major peak observed experimentally at 263 nm (π → π* electronic transition for benzene rings) occurs predominantly because of the transition from HOMO-5 to LUMO + 2. As the electron distribution for HOMO-5 lies largely at the peripheral benzene rings and is more concentrated on the center for LUMO + 2, it can be attributed to the charge transfer process. The UV–vis peak at 307 nm emerges due to the dominant transition from HOMO-4 to LUMO as can been seen from the figure. The synthesized 2D-Azeno NSs powder was dispersed into different solvents to analyze the phenomena of solvatochromism. As observed from Figure 3b, the 2D-Azeno NSs showed an intense green fluorescence at emission wavelength 501 nm on dissolving in DMSO, whereas it shows a slightly blue fluorescence in all other solvents. The UV light-driven images of the solution of 2D-Azeno NSs in various solvent media has been provided in Figure 3b (inset), showing the green fluorescence in DMSO, whereas a slightly bluish fluorescence in all other solvents. This is because DMSO fully solvates the 2D-Azeno NSs; however, all other solvents give suspensions where molecules might be aggregated. DFT analysis was performed to understand the fluorescence behavior of 2D-Azeno NSs under different solvent conditions. The observations concluded that after optimization in the ground state and excited state for all the solvents (Figure S3), the excited state HOMO–LUMO energy gap only reduced in the case of DMSO, while it was exactly the same for the other solvents. As can be observed from the PL spectra and UV images, the 2D-Azeno suspensions in other solvents also show bluish fluorescence, which can be attributed to the suspended or aggregated states. As the single-molecule DFT solvent models do not include the aggregated or suspended states, the single-molecule HOMO–LUMO gap does not change in other solvents, and experimental results for all other solvents are different from the computed model. The HOMO–LUMO energy gap in DMSO changed significantly from 3.45 eV (ground-state energy gap) to 2.86 eV (excited-state energy gap) (Figure 3d), leading to longer emission wavelength (red shift) corresponding to lower energy. DMSO being a polar aprotic solvent with very high dielectric constant stabilizes the charge-separated excited state more effectively than the ground state reducing the energy gap more as compared to the other solvents (Figure 3c). As emission at longer wavelength is considered to be more reliable for sensing applications and the synthesized material also showed a better solubility in DMSO in comparison to the other solvents, therefore, DMSO was chosen as the suitable solvent for further analysis and throughout the sensing application. The PL emission spectra for control experiments including the wavelength optimization and the fluorescence emission of the precursors and 2D-Azeno NSs have been shown in Figure S4. On wavelength optimization, the fluorescence emission was observed to be maximum at an excitation wavelength of 400 nm (Figure S4a), showing a PL quantum yield of approximately 34% in reference to Quinine sulfate dihydrate. The starting material pyromellitic diimide does not shown any emission, and *m*-phenylenediamine shows an emission at 535 nm, whereas the synthesized 2D-Azeno NSs showed an emission at 501 nm as demonstrated in Figure S4b. The stability and storage of the prepared 2D-Azeno NSs under different conditions were also analyzed to understand the effect of surroundings on its optical properties (shown in Figure 3e,f). 2D-Azeno NSs when subjected to a range of temperatures and to prolonged UV light exposure showed a significant stability (UV light-driven images in the inset) as presented in Figure 3e. For further storage of 2D-Azeno NSs, the prepared solution was kept under ambient light and in dark to observe the fluorescence behavior, and it was observed that the fluorescence intensity remains intact both under dark and light conditions with minute fluctuations; hence, the final solution of 2D-Azeno was stored in the dark throughout the studies.

**Figure 3:**
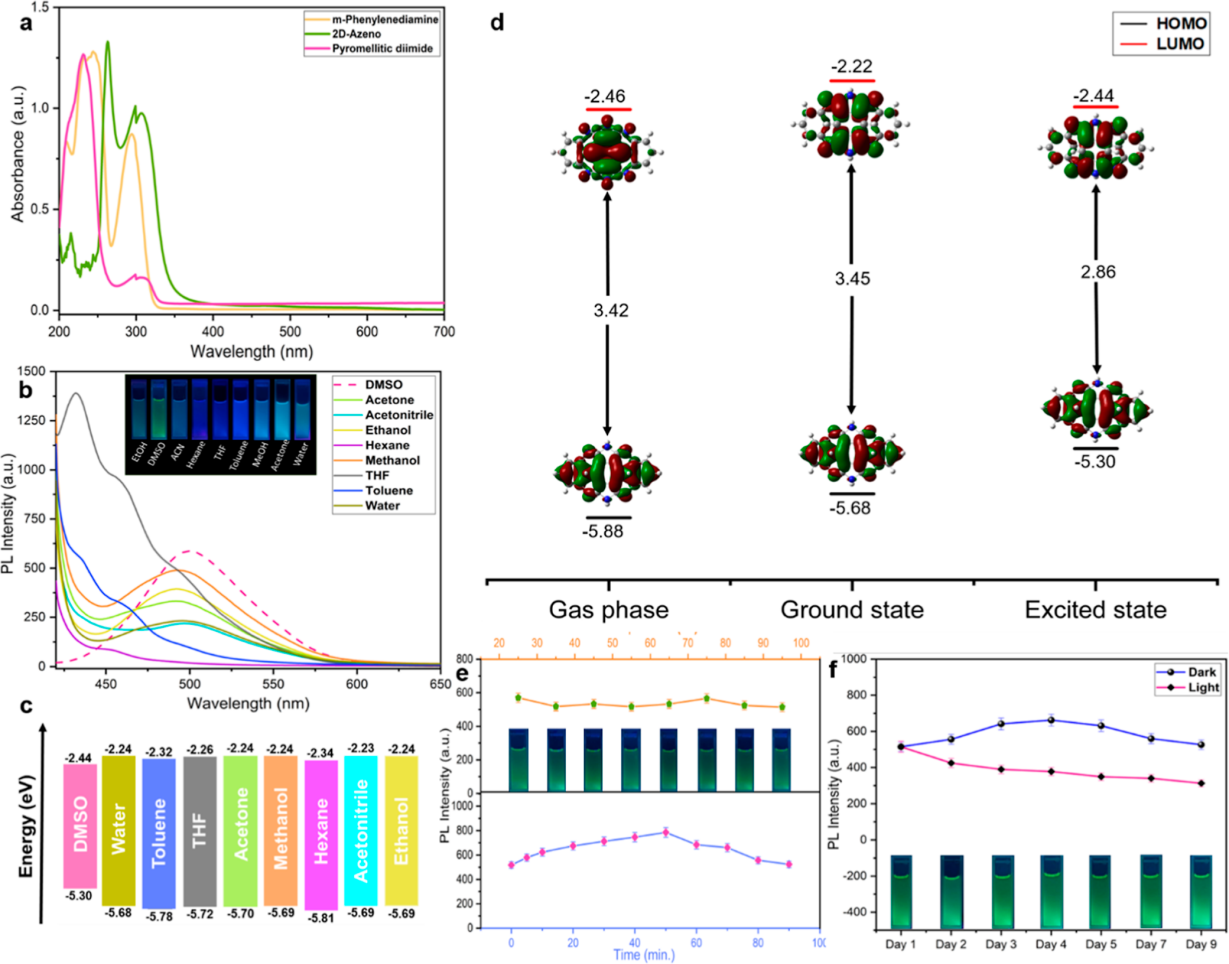
Optical properties of 2D-Azeno NSs. (a) UV–vis spectra for 2D-Azeno and its precursors. (b) PL spectra of 2D-Azeno in different solvents (the inset shows UV light-driven images of 2D-Azeno on dispersion in different solvents). (c) HOMO–LUMO energy gap for 2D-Azeno in different solvents. (d) Solvent-modulated charge distribution of frontier molecular orbital (HOMO–LUMO) in the ground state and excited state. Stability of 2D-Azeno under (e) a range of temperatures and UV exposure. (f) Dark and light conditions.

### Sensor Performance

The efficient fluorescence emission at 501 nm and high stability under a range of temperature and UV exposure renders the as-prepared 2D-Azeno NSs as a potential candidate for sensing applications. The fabricated NSs display efficient green emission which gets quenched and undergoes a slight blue shift on exposure to a specific biogenic amine SPM. The UV data showing the interaction between 2D-Azeno NSs and amines has been provided in Figure 4a. As evidenced by the figure, the UV absorbance peak at 263 nm undergoes a slight red shift to 269 nm along with a decrease in the intensity of the UV peak at 307 nm and shift to 308 nm. Additionally, a new hump near 364 nm is observed on interaction with SPM, whereas no prominent changes in UV–vis spectra were observed in the presence of other amines. Collectively, all the changes in the UV spectra suggest the possibility of noncovalent interactions, more specifically the presence of intermolecular hydrogen bonding. SPM being a polyamine contains multiple sites to form hydrogen bonds, altering the UV spectra because of the stabilization of π → π* electronic transition. Because the ground state is more stabilized than the excited state, the energy gap between the ground state and the first excited state is reduced, leading to the observed red shift in the primary absorbance peaks.^28,29^ The changes in the major transitions after SPM addition computed by DFT calculations are given in Table S3. The UV light-driven images for the selectivity of 2D-Azeno NSs toward SPM are shown in Figure 4b (inset).

**Figure 4:**
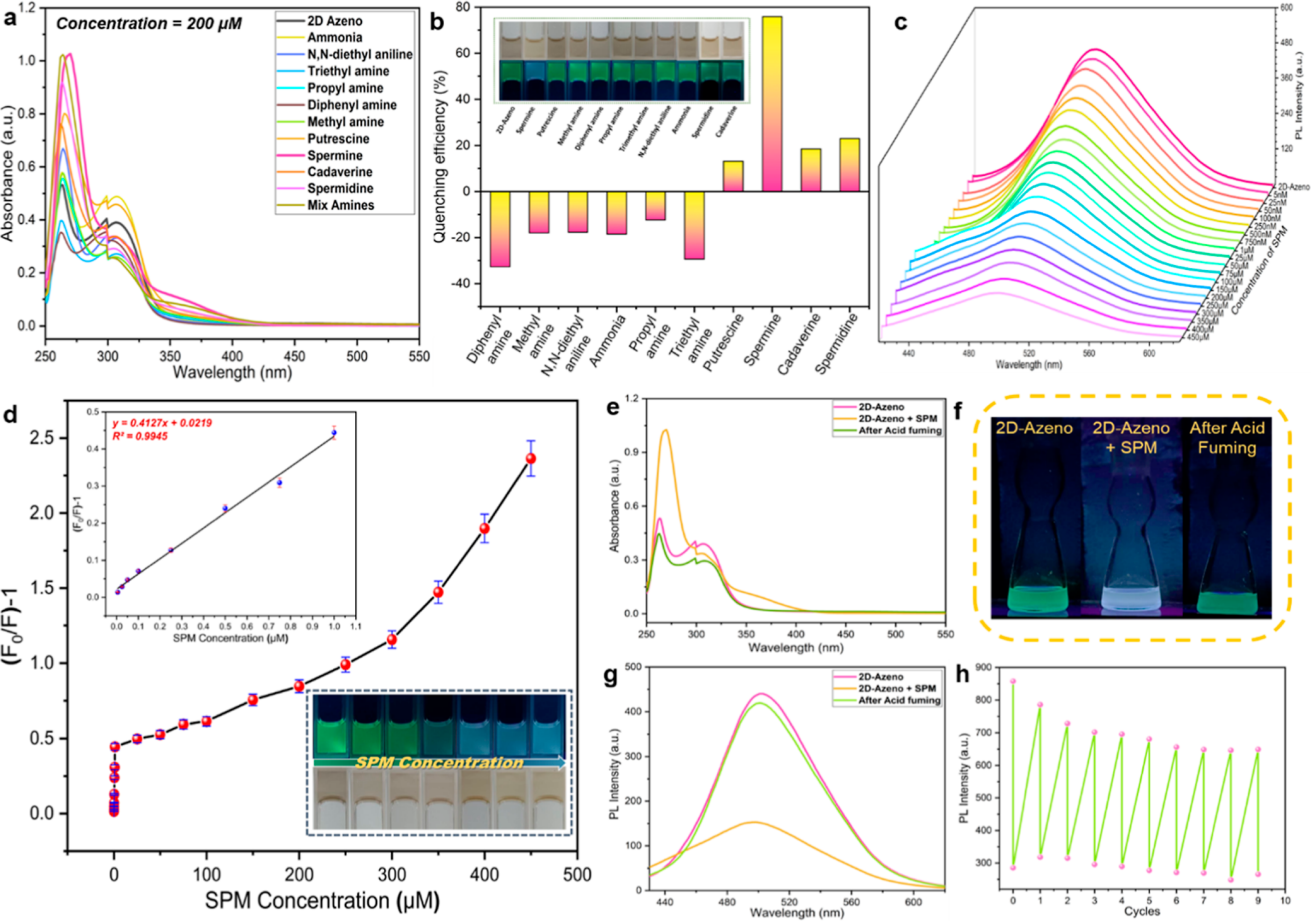
Sensing application of 2D-Azeno NSs. (a) UV–vis spectra of 2D-Azeno in the presence of several biogenic amines. (b) Selectivity of 2D-Azeno toward SPM (the inset shows the daylight and UV light-driven images of 2D-Azeno in the presence of biogenic amines). (c) Concentration-dependent study for SPM sensing. (d) Stern–Volmer plot for SPM detection (the inset shows the linear plot at low concentration, daylight, and UV light-driven images for 2D-Azeno NSs). Sensor reusability after acid fuming. (e) UV–vis spectra. (f) UV light-driven images. (g) PL emission spectra. (h) Sensor reusability for 9 cycles.

The fabricated 2D-Azeno NSs show significant selectivity toward SPM (Figures 4b and S5) along with the quenching efficiency of about 76%, further proving its applicability as a SPM sensor. The observed response can be accounted for by the multiple hydrogen bonding interactions between the polyamines and the symmetric 2D-Azeno probe. SPM having the longest aliphatic chain and a greater number of amine groups can undergo multiple hydrogen bonding interactions with the C–N and N–H sites of the probe, leading to the strongest fluorescence quenching, whereas other polyamines might create fewer or less favorable interactions, resulting in progressively weaker responses as observed. The concentration-dependent studies and the regression value calculations done using Stern–Volmer equation as shown in Figure 4c,d revealed a linear relationship at lower concentrations, within the linear range of 5 nM to 1 μM (Figure 4d inset) corresponding to a low detection limit value of around 4.35 nM (0.88 ppb). When viewed under the UV lamp, the fluorescence of the 2D-Azeno HICTM sensor was substantially quenched along with a trivial blue shift as suggested by Figure 4d (inset). The shift in the green fluorescence toward blue fluorescence was further confirmed by the CIE 1931 color image chromaticity diagram, showing that the chromaticity coordinates (*x*,*y*) changed from (0.176, 0.442) to (0.181, 0.347) as can be seen from Figure S6. The sensing performance of the developed 2D-Azeno NS has been further examined by comparing it to the already reported sensing techniques for SPM detection (Tables 1, S4, and S5). Compared with conventional HPLC and other commercially available techniques, this work based on the fluorescence method presents a simpler workflow and faster readout, whereas chromatographic methods retain an advantage in resolving complex matrices but are tedious to operate.

**Table 1:** Comparison Table Showing a Comparison of Previously Published Literature for SPM Detection and the Developed 2D-Azeno NSs-Based Sensor

probe	method of detection	concentration range (μM)	real sample	LOD (μM)	recovery (%)	ref
PPDD@SC4AD	Fluorescence sensor (Turn-On)	0–100	Tumor cells	0.94	-	^30^
EuMOF-FITC composite	Fluorescence sensor	0–395.37	Fish samples	5.48	-	^15^
d-CNPs/G-QDs	Fluorescence sensor	2–40	Shrimp & pork Samples	0.33	100–102.52 and 99.25–103.73%	^31^
CB@AG	Fluorescence sensor	6–2500	Human urine and blood	6.00	-	^22^
PFBT-MI	Fluorescence sensor	1–10	Urine sample	0.33	105–107%	^32^
P1QDs and P2QDs	Fluorescent sensor	0.05–15 and 1–12	Human serum	0.29 × 10^–2^ & 1.66 × 10^–3^	96.88–106.90%	^23^
**2D-Azeno**	**Fluorescent sensor**	0.005–450 μM	**Human serum and urine**	**4.35 × 10** ^**–3**^ **(0.88 ppb)**	**97–102%**	**This Work**

The comparison suggests that the proposed sensor showed either better or comparable detection limits to the previously reported sensors. The impedometric sensor reported by Luhar et al. showed a better detection limit, but the impedometric sensors have complex handling and are more prone to interference as compared to fluorescence sensors. Additionally, the reusability of the sensor being a crucial parameter for the practical applications was tested as evidenced by Figure 4e–h. As suggested, the 2D-Azeno HICTM sensor could be reactivated and regenerated by using acid (HCl) fuming. SPM, due to the presence of amine groups, participates in strong intermolecular hydrogen bonding with 2D-Azeno NSs. On exposure to fuming HCl, the amine groups of SPM undergo protonation, resulting in the weakening or disruption of intermolecular H-bonding leading to the restoration of excited-state transitions and fluorescence regeneration (Figure 4e–g), suggesting the sustainability and robustness of the developed sensor for 9 consecutive cycles without any significant loss of performance as displayed in Figure 4h. The long-term stability of 2D-Azeno NSs after acid fuming has been monitored for 30 days, showing a stable UV–vis spectrum along with no significant changes in the PL emission intensity (Figure S7), retaining a good structural and photophysical stability after acid fuming.

The applicability of the sensor in real-world scenarios was monitored quantitatively for urine and blood serum as SPM is also a cancer biomarker, whereas qualitative monitoring has been performed using meat and seafood samples to determine the freshness. The 2D-Azeno NS-based probe exhibited a remarkable performance toward SPM detection in both the biological samples (urine and blood serum) with excellent recovery rates as shown in Table 2. In order to investigate the applicability and feasibility of the synthesized sensor in real-world scenarios, the anti-interference studies were performed using the amines and other entities present in the biological as well as food samples (Figure S8). The developed sensor demonstrated a high efficiency and anti-interference ability even in the presence of several biological compounds and ions showing its feasibility in real-world applications. The sensor’s practical endurance in complicated biological matrices is confirmed by reusability study (Figure 5a), where fluorescence signals remain measurable up to four regeneration cycles despite a modest decrease in intensity per cycle, highlighting the operational robustness of 2D-Azeno NSs (UV light images given in the Figure 5a inset).

**Table 2:** Recovery Table for Real Sample Analysis of SPM Sensing

real samples	concentration added (μM)	concentration detected (μM)	recovery (%)	RSD (%) (*n* = 3)
Urine	0.25	0.26	101.08	2.49
	0.75	0.77	102.17	2.65
	50.00	50.48	100.95	3.91
	150.00	148.32	98.88	5.22
	350.00	341.01	97.43	4.27
Blood Serum	0.25	0.26	100.85	1.72
	0.75	0.76	100.50	2.16
	75.00	74.49	99.32	2.73
	200.00	199.80	99.90	4.70
	350.00	346.36	98.96	5.12

**Figure 5:**
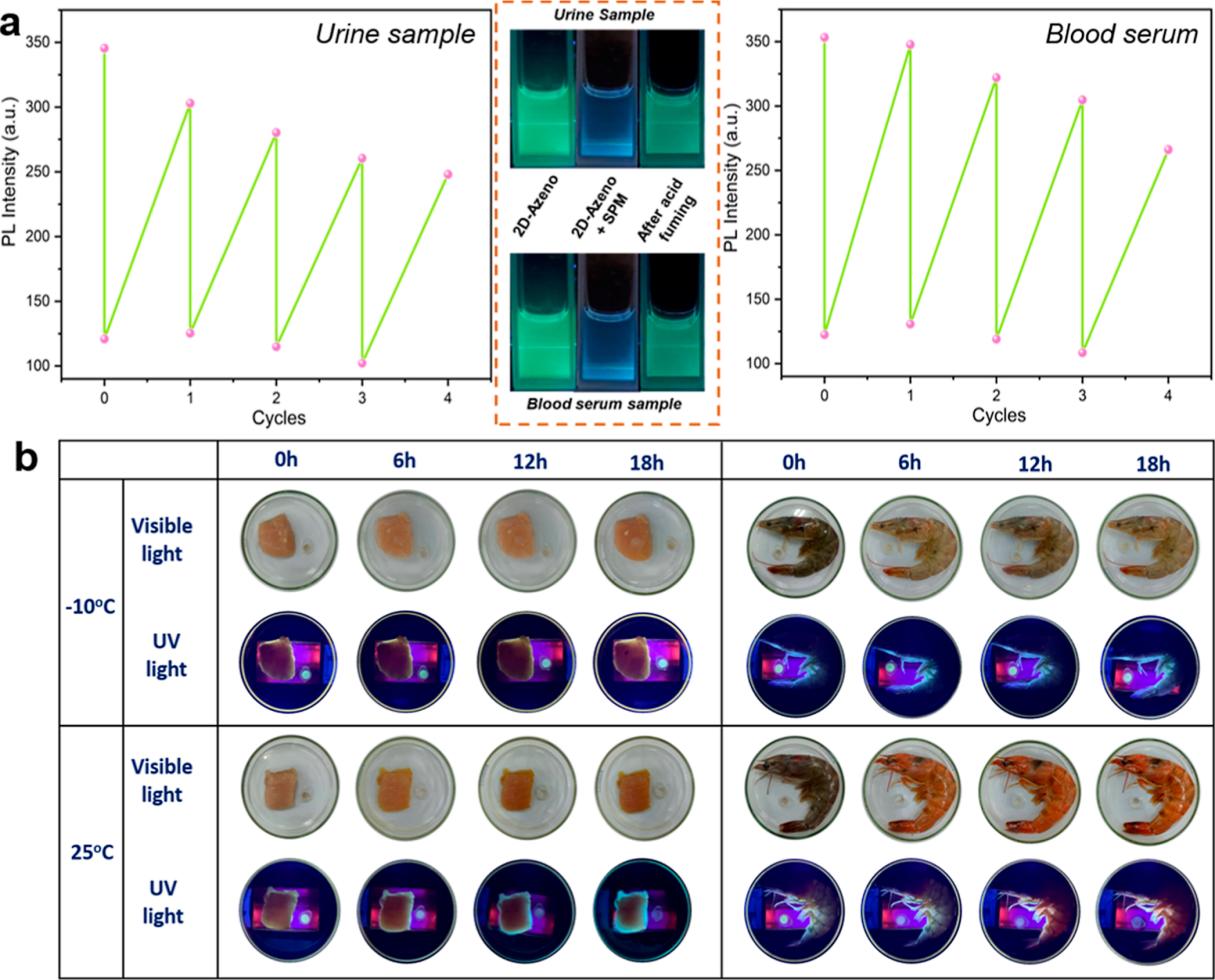
Real sample application of 2D-Azeno NSs. (a) Sensor reusability for SPM detection in biological samples including UV light-driven images before and after acid fuming. (b) Qualitative monitoring of meat and shrimp sample freshness when stored at different temperatures in the presence of 2D-Azeno NSs.

### Qualitative Monitoring of Food Freshness

SPM, a biogenic amine and an established biomarker for determining the food freshness alongside some other amines that are released during food spoilage, enables reliable monitoring of meat and shrimp quality. In order to determine the sensor feasibility in the presence of real food samples, the sensor solution was kept in the presence of meat and shrimp samples for 18 h at 25 °C and −10 °C. As illustrated in Figure 5b, the results showed that no visible fluorescence quenching or changes were observed across all the time plots (0, 6, 12, and 18 h) when the meat and shrimp samples were refrigerated, indicating the sensor stability and negligible SPM release under cold storage. On the contrary, the fluorescence gradually decreased for both the samples stored at 25 °C owing to the release of SPM vapors during the spoilage of food samples primarily due to the decarboxylation of free amino acids. As displayed in Figure S9, the fluorescence intensity of 2D-Azeno solution decreased with storage time for both chicken and shrimp samples kept at 25 °C, reflecting the continuous release of SPM. As the cellular degradations starts before the bacterial degradation due to autolysis, bound SPM is released into the free surface moisture of the meat losing its premium freshness, although visually appearing fresh. In contrast, samples stored at −10 °C showed only minor spectral variation, indicating the low temperature effectively suppressed the degradation process. These results validate the sensor’s selectivity and sensitivity for simple, immediate, qualitative/semiquantitative detection of endogenous SPM detection in perishable foods.

### SPM Detection Mechanism

To study the sensing mechanism for SPM detection, the excitation and emission spectra of 2D-Azeno NSs were overlapped with the UV–vis absorbance spectra of SPM. The data revealed that as SPM does not inherently absorb in the UV–vis region, the overlap between material excitation or emission and SPM absorbance is not possible consequently, ruling out the possibility of fluorescence quenching due to Inner Filter Effect (IFE) or Fluorescence Resonance Energy Transfer (FRET) as depicted by Figure S10a. The UV–vis spectra of 2D-Azeno NSs showing two major peaks at around 263 and 307 nm changed significantly after interaction with SPM. The UV peak at 263 nm shifted slightly toward longer wavelength and the intensity of the peak at 307 nm decreased, whereas a peak around 364 nm appeared after SPM sensing (Figure 6a), suggesting the possibility of an electrostatic interaction including hydrogen bonding. The development of a new peak sometimes also represents the proton transfer between the donor and acceptor, but this does not apply here because the solvent used throughout the studies is DMSO (a polar aprotic solvent). Hence, the interaction between the developed 2D-Azeno NSs and SPM is intermolecular hydrogen bonding. This has been further backed up by the zeta potential data as presented in Figure S10b. The zeta potential for 2D-Azeno NSs was observed to be slightly negative (−0.24 eV), which shifted to a positive value of 1.14 eV on addition of SPM due to the change in dipole effects. Additionally, the XRD data after adding SPM to the 2D-Azeno NSs also revealed that the crystal structure of 2D-Azeno remained intact with minute shifts (0.02° to 0.2°) in the major peaks, suggesting the slight distortion in the lattice without any disruption in the long-range order supporting noncovalent interactions or hydrogen bonding (Figure 6b). In order to prove the quenching mechanism due to intermolecular H-bonding, DFT studies were done before and after adding SPM as shown in Figure 6d. Initially, the natural bond orbital analysis was done using DFT in order to find the most probable site for H-bonding as the 2D-Azeno structure contains six nitrogen atoms. The charge present on all the atoms has been shown in Figure S11, and the interpretation for N atoms along with the atom number has been provided in Table S6. The NBO data revealed that both in the gas phase and after stabilization in the solvent medium (DMSO), the N21 and N23 atoms have the greater charge and lone pair density and they can act as the primary H-bond acceptors. But as can be seen from the structure of 2D-Azeno, both N21 and N23 have a hydrogen atom attached to them, carrying a positive charge due to the high negative charge on N21 and N23; therefore, these sites can act as H-bond donors toward SPM, whereas the electron density for N27–N30 might also participate in conjugation, leading to a decreased charge and secondary H-bond participation probability due to H-bond accepting from SPM. The H-bond accepting and donating sites has been further confirmed by the electrostatic potential map for 2D-Azeno NSs (shown in the Figures 6a inset and S11c) where the red regions represent the high electron density and H-bond acceptor sites (N27–N30), whereas the blue regions generally represent the electron-deficient sites having H-bond donating capability. As can be seen from the HOMO–LUMO frontier orbital distribution of 2D-Azeno NSs (Figure 6d), the hole density is mainly located on the peripheral benzene rings, and the electron density is distributed across the central benzene and the pyrrolidine core, leading to an intramolecular charge transfer (ICT) between the donor (peripheral benzene rings) and acceptor (central benzene and pyrrolidine) unit on excitation. On addition of SPM, the HOMO–LUMO energy gap becomes wider (see Figure 6) as compared to bare 2D-Azeno NSs, corresponding to the decrease in ICT in the emissive excited state and increased quenching effect along with slight hypsochromic shift in emission. For the intermolecular hydrogen bonding interaction, the bond lengths for 2D-Azeno NSs and SPM interaction were of the order of 1.7 to 2.0 A^o^. Additionally, the XPS spectra shown in the Figure 6d inset for 2D-Azeno NSs before and after SPM addition do not undergo any significant changes, except slight shifts toward higher binding energy values. As shown in the Figure, the peaks in N 1s spectra shift from 400.26 to 401.59 eV (for N=C) and from 399.67 to 399.79 eV (for C–NH), whereas the peaks for C 1s spectra shift from 284.8 to 284.97 eV, 285.06 to 285.64 eV, and 288.26 to 288.53 eV for N–C=N, C=N, and C=C/C–C, respectively. These slight shifts in the XPS binding energy strongly support the intermolecular hydrogen bonding as C–NH acts as a hydrogen bond donor toward SPM, whereas the N atoms in C=N can act as the hydrogen bond acceptor toward SPM, altering the electron density and delocalization of electrons. The lifetime measurements shown in Figure 6c further support the explained mechanism as the TRPL lifetime 2D-Azeno NSs decreased from τ = 4.55 ns to τ = 2.93 ns after SPM addition, confirming the alteration of the excited-state deactivation pathway and enhanced nonradiative relaxation. Collectively, the SPM sensing via the 2D-Azeno HICTM sensor occurs because of all the above-mentioned spectral changes after SPM addition along with HOMO–LUMO widening, indicating a quenching mechanism due to the formation of a nonemissive 2D-Azeno NSs-SPM complexation via intermolecular hydrogen bonding.

**Figure 6:**
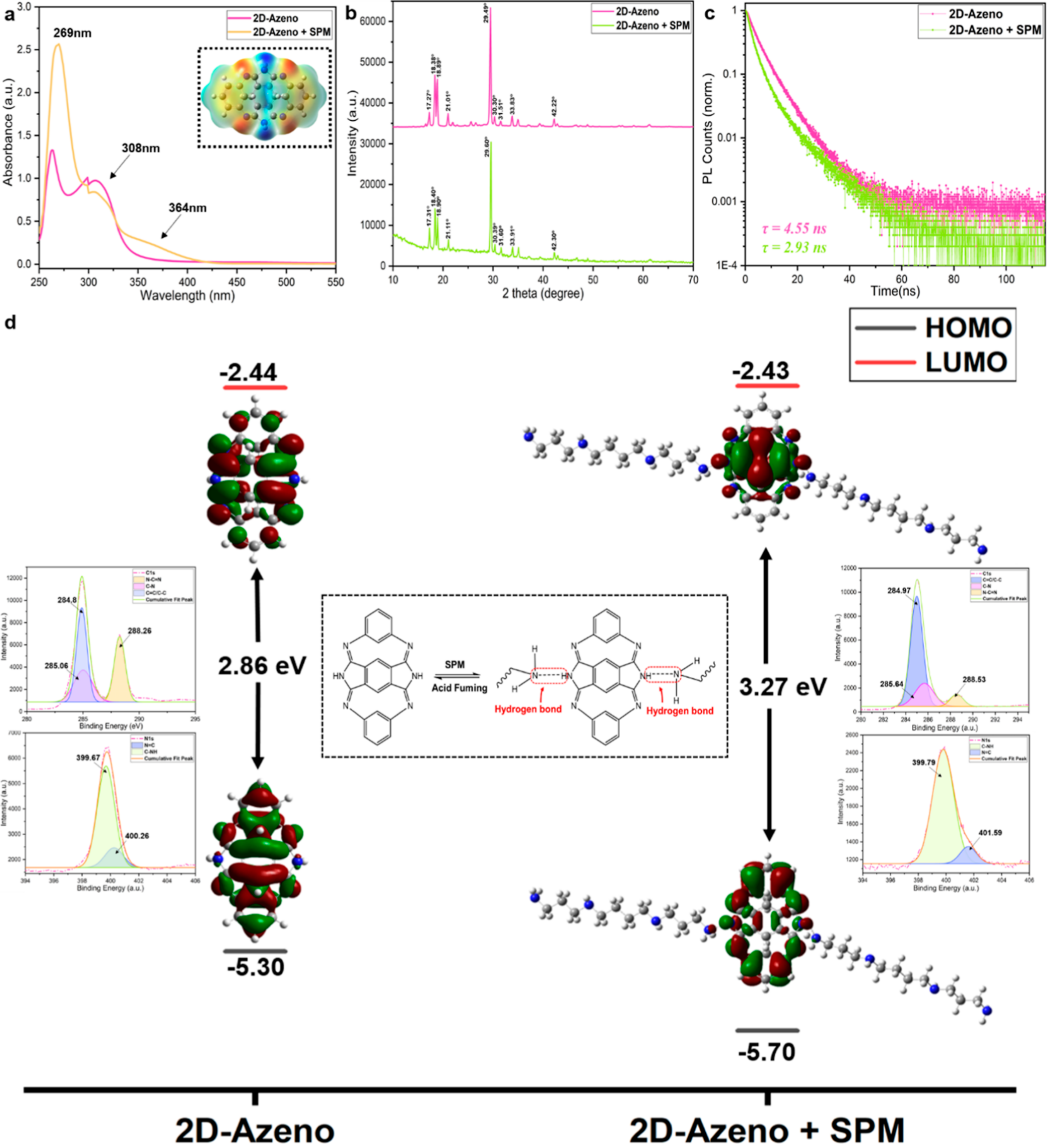
Mechanistic insights for SPM detection. (a) UV–vis spectra (the inset shows the electrostatic potential (ESP) map for 2D-Azeno). (b) XRD analysis. (c)­Time-resolved photoluminescence­(TRPL) spectra for 2D-Azeno before and after SPM addition. (d) HOMO–LUMO energy gap and frontier orbital distribution after adding SPM (the inset showing XPS spectra after SPM sensing).

## Conclusion

This work establishes 2D-Azeno nanosheets as a robust, reusable, and mechanistically well-resolved platform for selective SPM detection across diverse analytical environments. We have introduced a truly intrinsic fluorescent probe 2D-Azeno nanosheets derived from a facile Schiff-condensation of *m*-phenylenediamine and pyromellitic diimide, followed by ultrasonic exfoliation. The layered π-conjugated architecture, enriched with hydrogen-bond-active amine sites, enables highly specific supramolecular recognition that perturbs the excited-state electronic structure, producing ratiometric quenching and a diagnostic blue-shifted emission. TD-DFT analyses elucidate the molecular origin of this response, revealing site-selective hydrogen bonding that suppresses intramolecular charge transfer and increases the frontier orbital energy gap. This mechanistic clarity combined with reversibility upon protonation, sustained photostability, and resistance to matrix-derived interferences yields a sensing platform with both practical robustness and theoretical grounding. Successful validation in human serum, urine, and spoilage-prone meat systems underscores the translational potential of 2D-Azeno HICTM sensor for next-generation food safety monitoring and biomedical diagnostics, positioning this material framework as a powerful model for future 2D organic sensing architectures.

## Supplementary Material



## Notes

TEM images of bulk-Azeno; AFM and EDS spectra; basis sets and TD-DFT functionals used for the theoretical calculations; UV–vis spectral transitions for 2D-Azeno before and after SPM interaction; HOMO–LUMO energy gap for 2D-Azeno NSs in different solvent media, PL spectra for optimization, control experiments, and selectivity studies; chromaticity diagram; comparison table; anti-interference study; NBO analysis for SPM interaction; mechanism analysis including zeta potential; NBO charge distribution map and ESP map; and LOD calculations (PDF)
