# Supplementary material for: Supramolecular ICT Modulation in π‑Extended 2D-Azeno Nanosheets for Reversible Fluorescence Sensing of Spermine at the Sub-10 nM Level
Source: Anal Chem. 2026 Jul 31;98(31):22902–13. doi: 10.1021/acs.analchem.6c02145 (PMC13470972; doi:10.1021/acs.analchem.6c02145)
Supplement: Supplementary file 1 [file ac6c02145_si_001.pdf]

Supporting Information

Supramolecular ICT modulation in π-Extended 2D-Azeno Nanosheets for Reversible Fluorescence Sensing of Spermine at the Sub-10 nM Level

Priyanka Rana[1], Deepak Dabur[1], Hui-Fen Wu*[1, 2, 3,4,5,6]

[1]Department of Chemistry, National Sun Yat-Sen University, Kaohsiung, 70, Lien-Hai Road, Kaohsiung, 80424, Taiwan

[2]School of Pharmacy, College of Pharmacy, Kaohsiung Medical University, Kaohsiung, 807, Taiwan

[3]Institute of Medical Science and Technology, National Sun Yat-Sen University, Kaohsiung, 80424, Taiwan

[4]School of Medicine, College of Medicine, National Sun Yat-Sen University, Kaohsiung, 80424, Taiwan

[5]Institute of Precision Medicine, National Sun Yat-Sen University, Kaohsiung, 80424, Taiwan.

[6]Institute of Biopharmaceutical Science, National Sun Yat-Sen University, Kaohsiung, 80424, Taiwan

*Corresponding author: Prof. Hui-Fen Wu (Department of Chemistry, National Sun Yat-Sen University)

Email: hwu@faculty.nsysu.edu.tw

Phone: +886-7-5252000-3955; Fax: +886-7-5253909

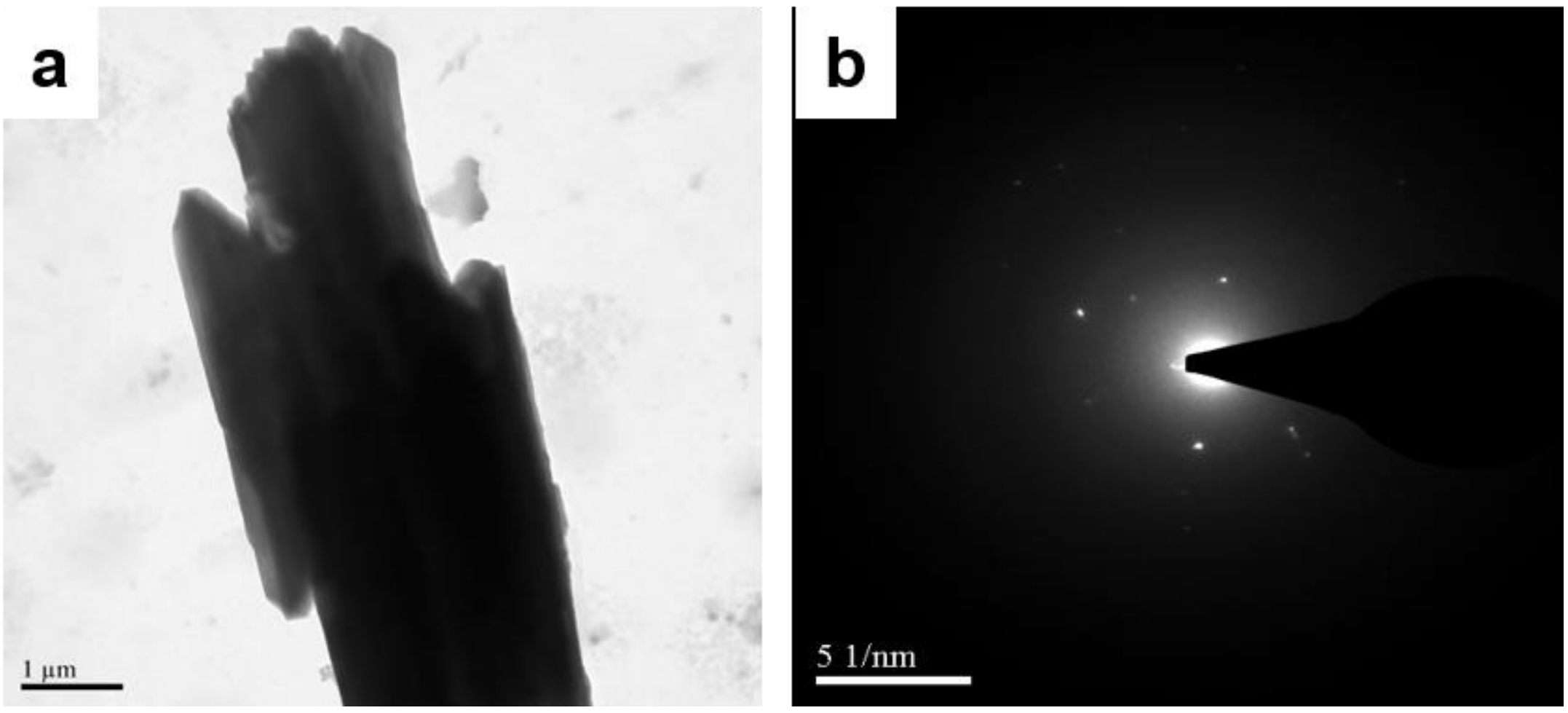

Figure S1. (a) Transmission electron microscope (TEM) image for Bulk-Azeno **(b)** SAED pattern for Bulk-Azeno.

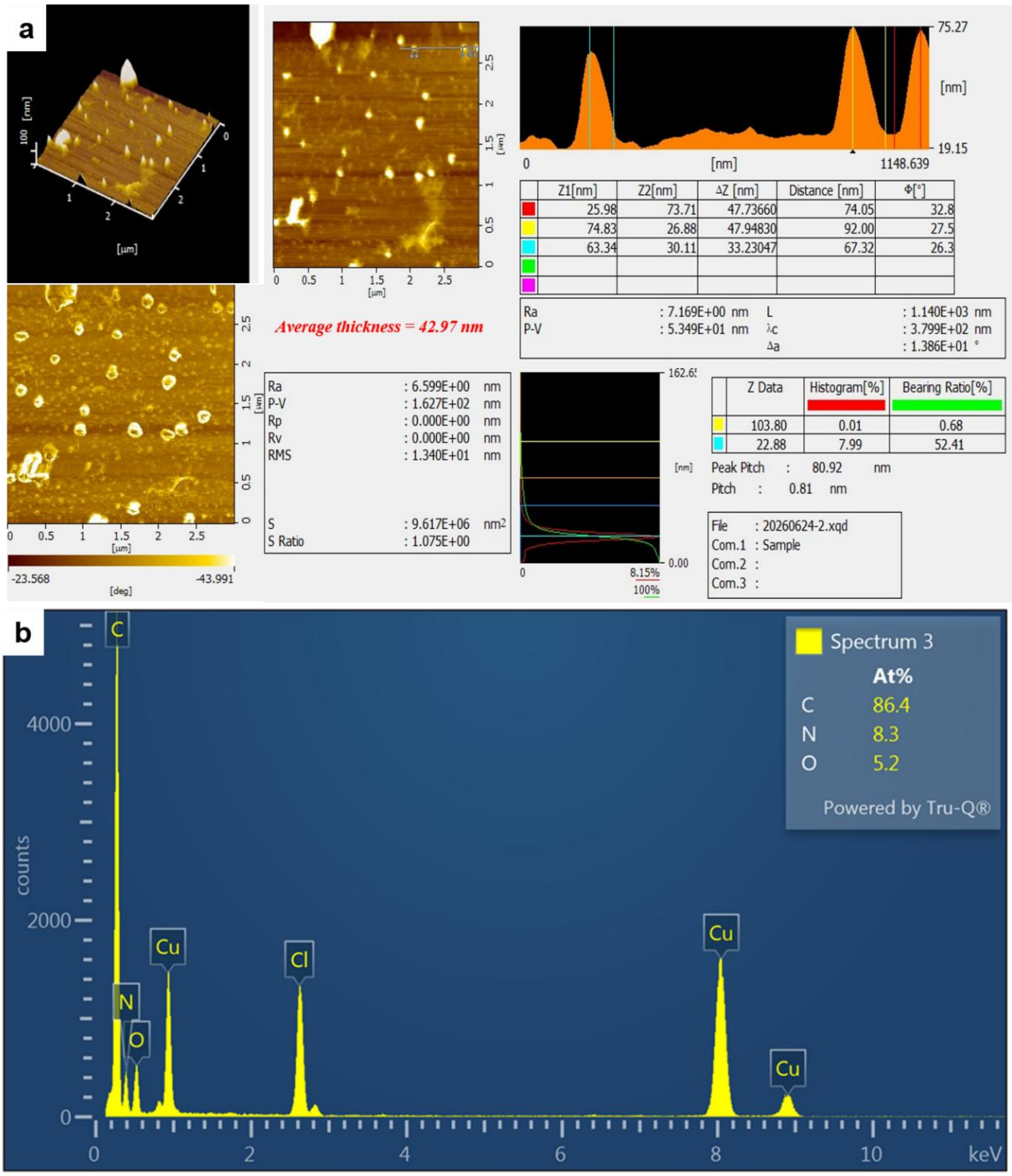

Figure S2. (a) Atomic Force Microscopy (AFM) images and thickness height profile for 2D-Azeno NSs **(b)** Energy Dispersive X-ray Spectra (EDS) showing the elemental distribution of 2D-Azeno NSs.

Table S1. Basis sets and TD-DFT functionals used for the theoretical calculations using Gaussian 16W

**Calculation**	**Calculation Method**	**Basis Set**	**Solvation**
Solvatochromism	B3LYP	6-31(d,p)	None
			DMSO
			Water
			Toluene
			THF
			Acetone
			Methanol
			Hexane
			ACN
			Ethanol
NBO	B3LYP	6-31(d,p)	None
			DMSO
Mechanism	B3LYP	6-31(d,p)	DMSO

Table S2. UV-Vis spectral transitions for 2D-Azeno NSs in DMSO.

Transition		Coefficient	Energy (eV)	Wavelength (nm)	Oscillator strength	Expt. (nm)
from	to					
HOMO-8 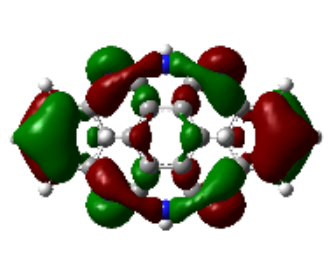	LUMO+1 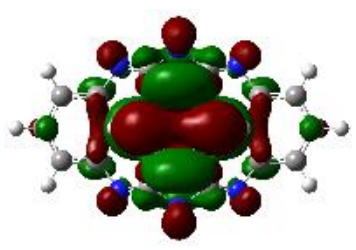	21.42%	4.6661	265.71	0.2774	263nm
HOMO-5 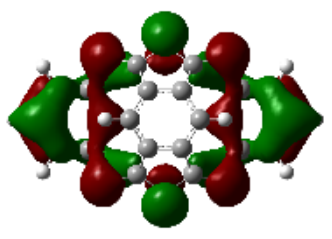	LUMO+2 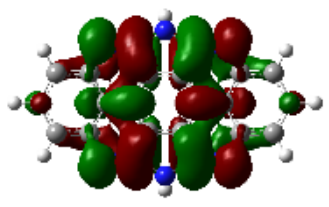	23.63%				
HOMO 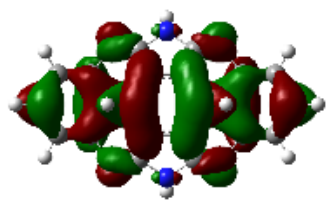	LUMO+7 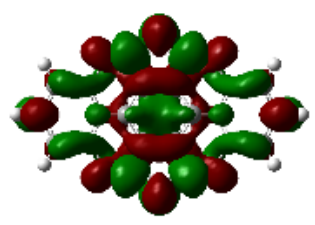	1.00%				
HOMO-7 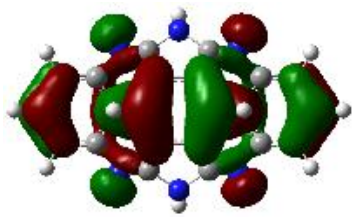	LUMO+1 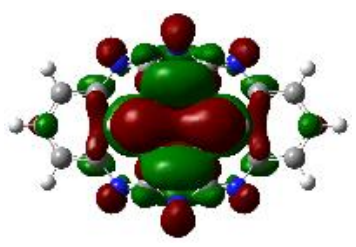	1.11%	4.0345	307.31	0.3138	307nm
HOMO-6 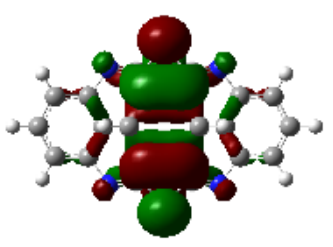	LUMO 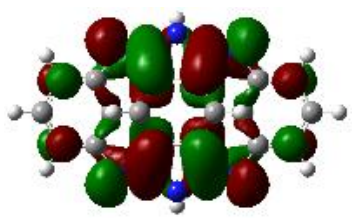	13.09%				
HOMO-5 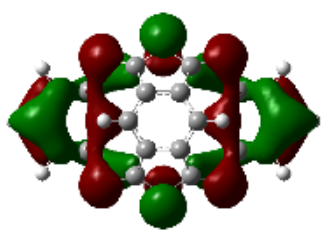	LUMO+2 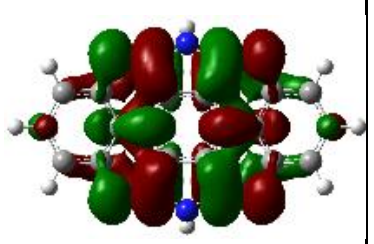	3.87%				
HOMO-4 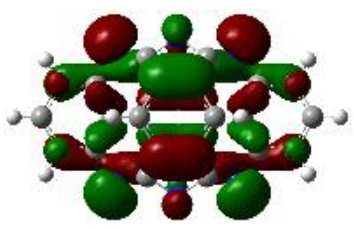	LUMO 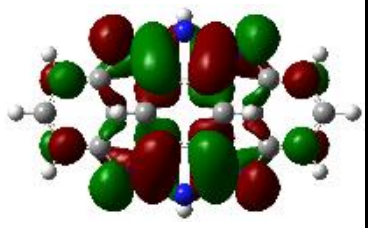	22.76%				
HOMO-3 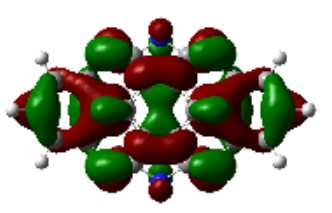	LUMO+2 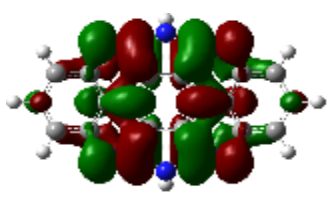	6.58%				

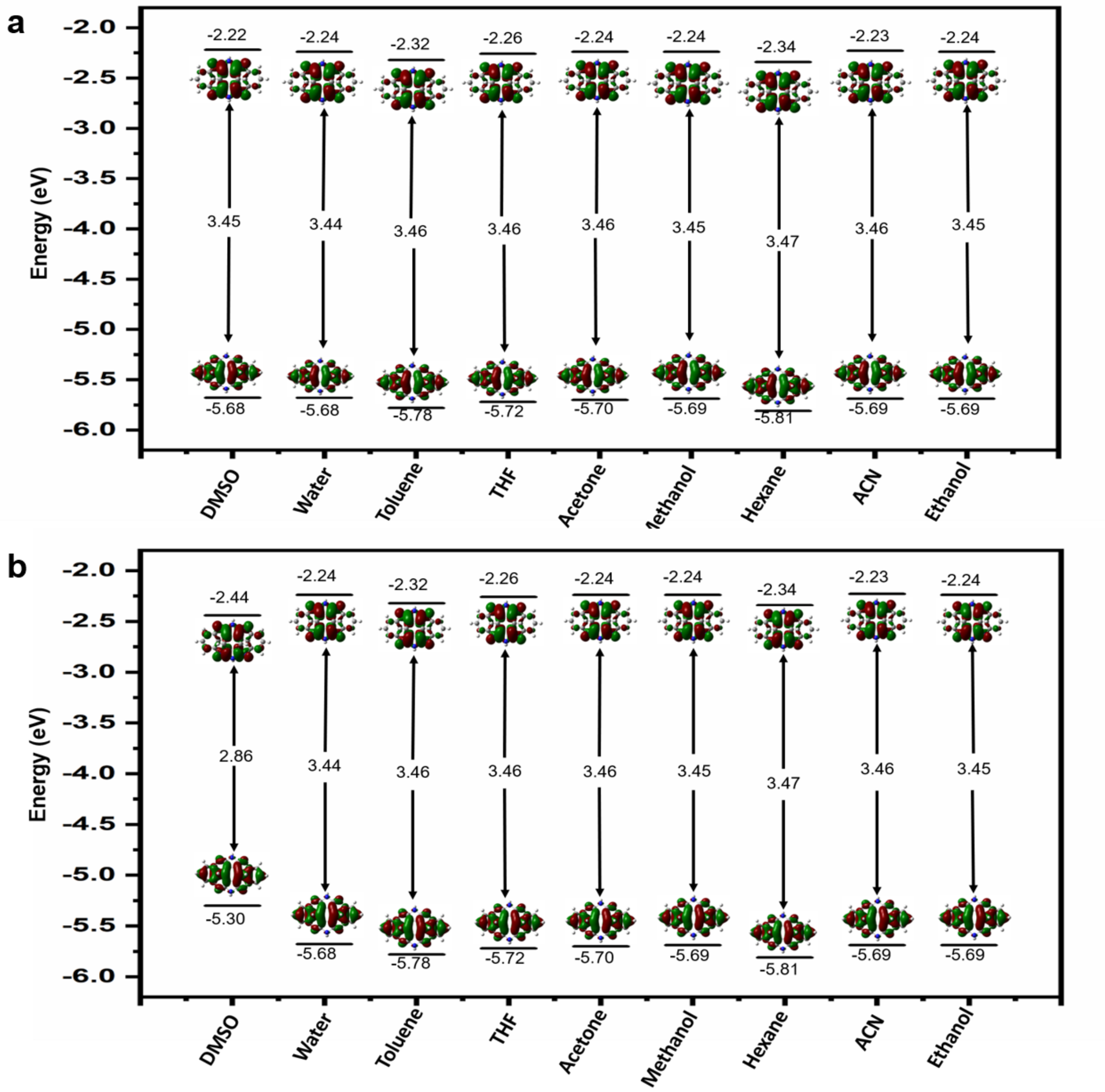

Figure S3. HOMO-LUMO energy gap for 2D-Azeno NSs in different solvent mediums **(a)** in Ground state **(b)** in Excited state

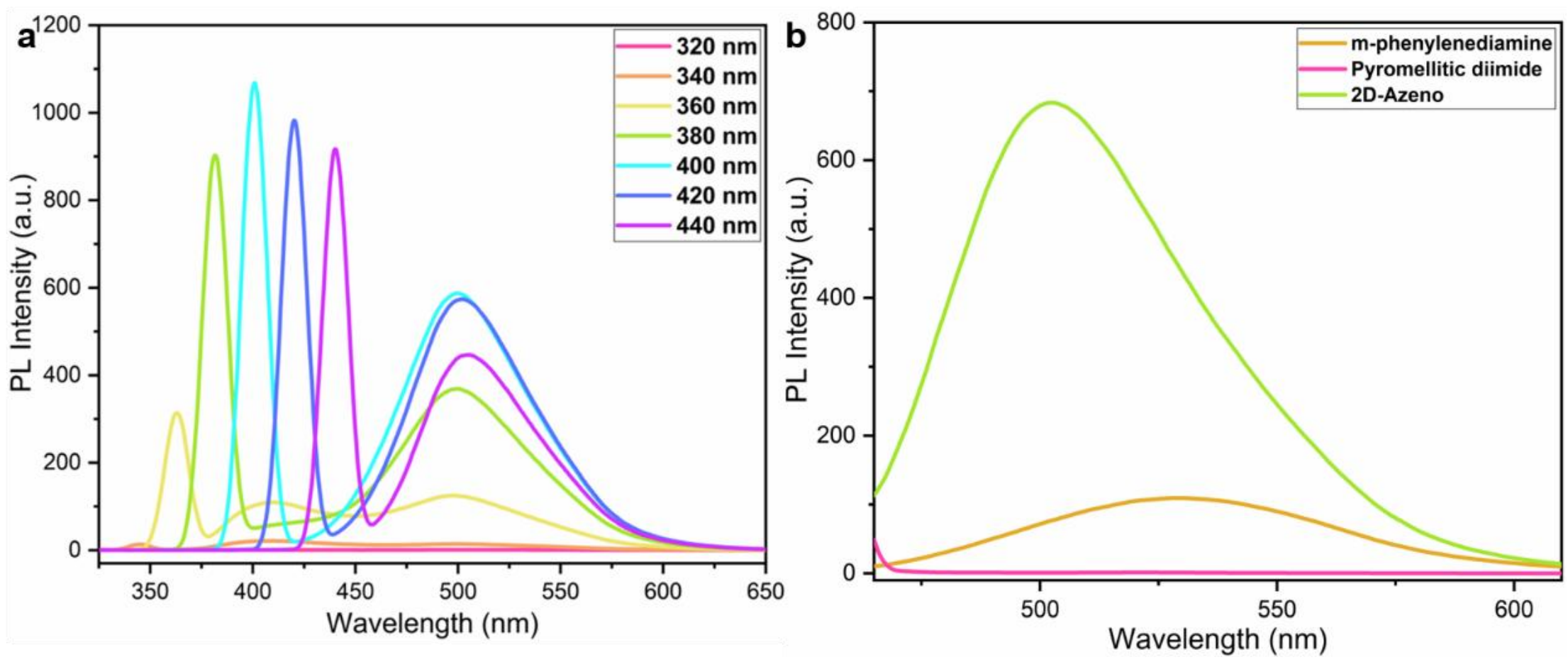

Figure S4. (a) Excitation dependent PL emission spectra **(b)** PL emission spectra of precursors and 2D-Azeno NSs.

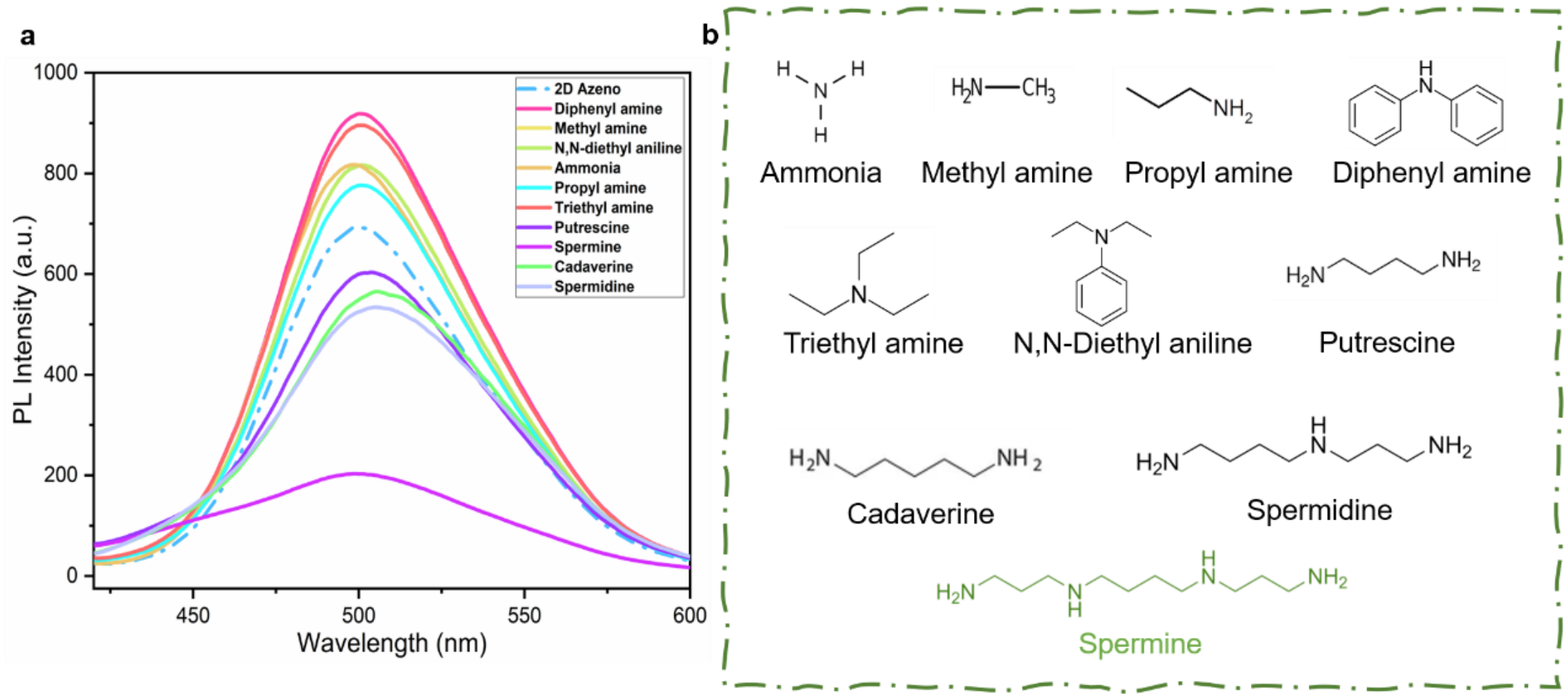

Figure S5. (a) PL spectra showing the selectivity study for the sensing of biogenic amines **(b)** Structures of different biogenic amines.

Table S3. UV-Vis spectral transitions for 2D-Azeno HICTM sensor after interaction with SPM.

Transition		coefficient	Energy (eV)	Wavelength (nm)	Oscillator strength	Expt. (nm)
from	to					
HOMO-16	LUMO	1.26%	4.5909	270.07	0.1308	269nm
HOMO-15	LUMO	3.90%				
HOMO-13	LUMO+2	7.35%				
HOMO-12	LUMO+2	32.19%				
HOMO-11	LUMO+2	1.86%				
HOMO-5	LUMO	46.33%	4.0140	308.88	0.0015	308nm
HOMO-3	LUMO	2.45%				
HOMO-14	LUMO	9.43%	3.4377	360.66	0.0224	364nm
HOMO-12	LUMO	9.57%				
HOMO-11	LUMO	2.07%				
HOMO-10	LUMO	14.69%				
HOMO-1	LUMO+2	10.01%				

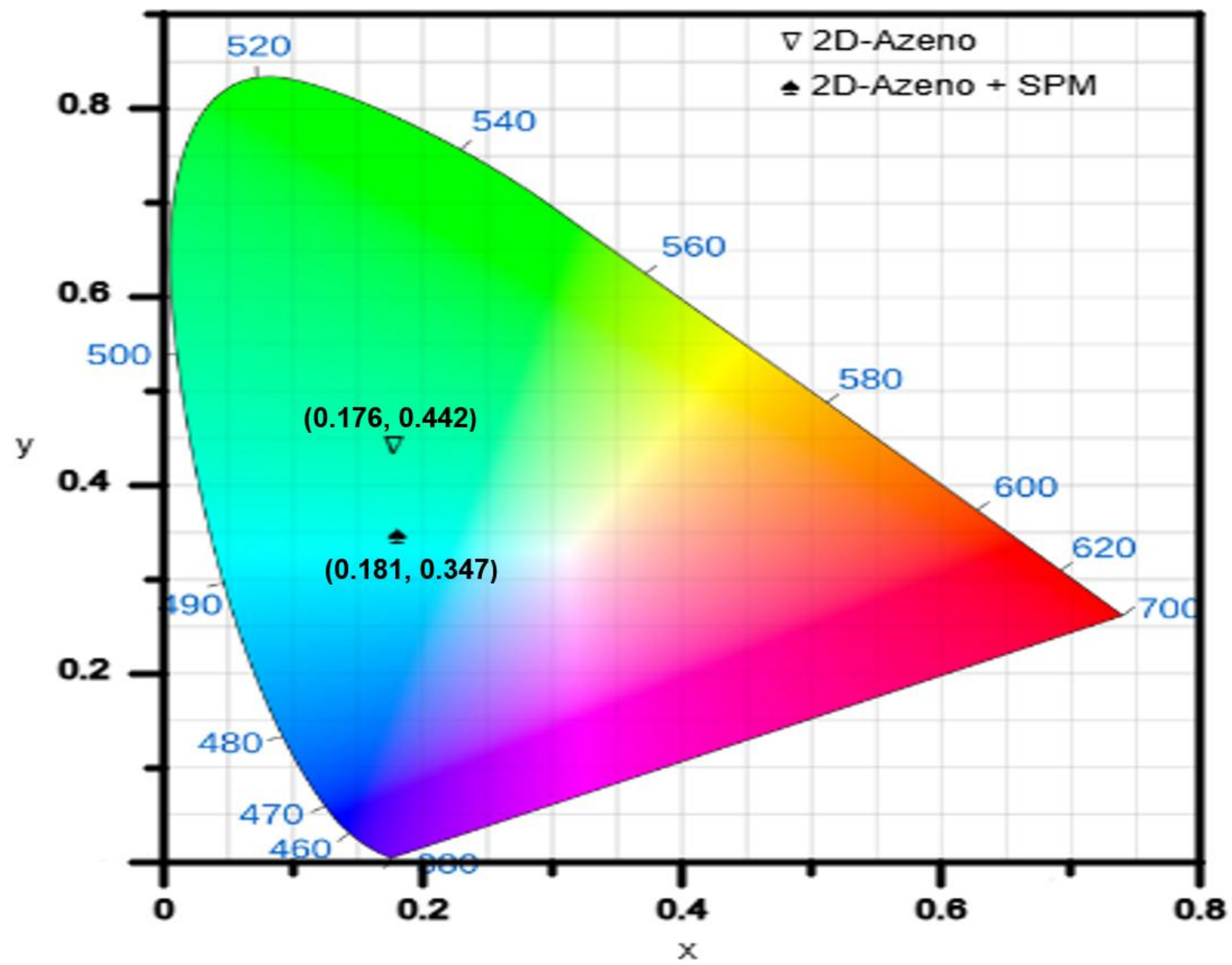

Figure S6. CIE chromaticity diagram before and after addition of SPM.

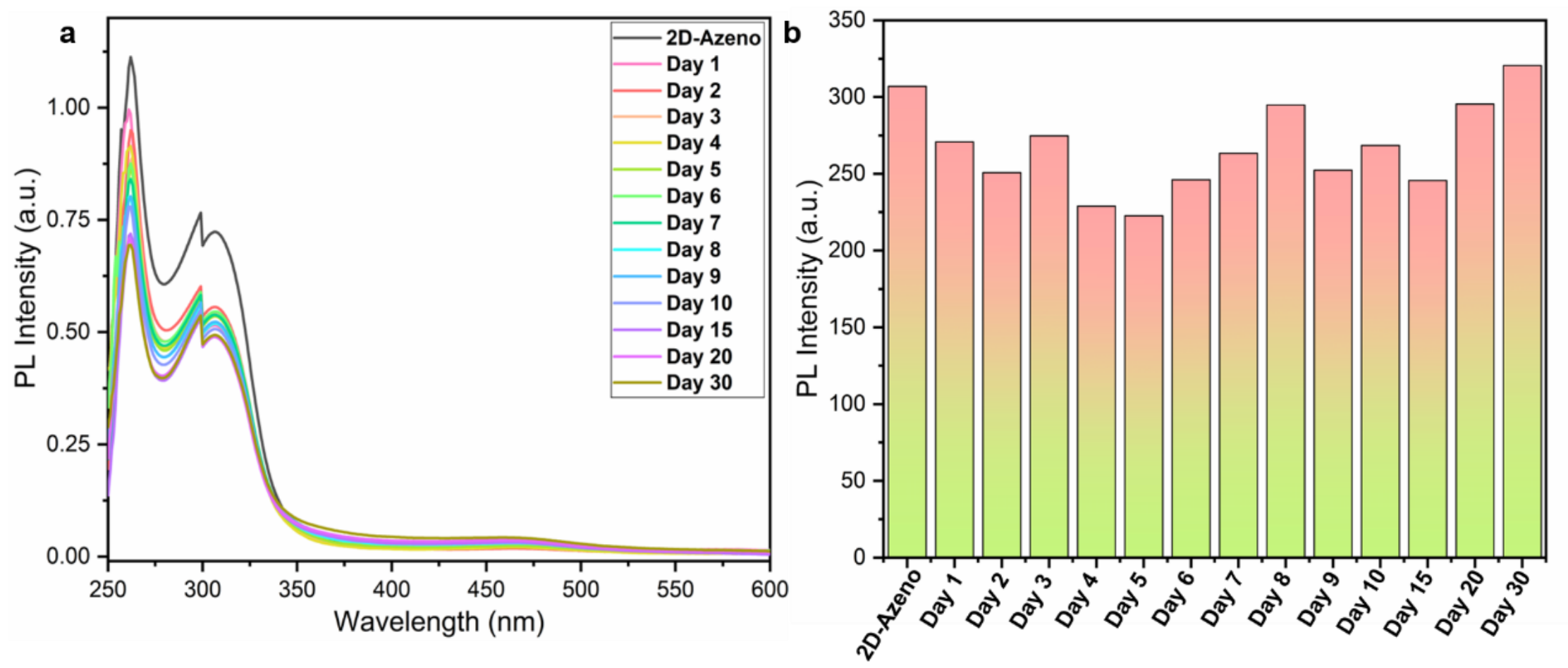

Figure S7. Stability of 2D-Azeno NSs after acid fuming (a) UV-Vis spectra (b) Bar graph showing PL emission intensity.

Table S4. Comparison table showing a comparison of previously published literature for SPM detection and the developed 2D-Azeno HICTM sensor.

Probe	Method of detection	Concentration range (µM)	Real Sample	LOD (µM)	Recovery (%)	Ref.
Boronic acid @ plastic chip	Impedometric sensor	4.9×10^{-4} - 4.94	Urine and blood plasma	1.00×10^{-5}	-	1
AuNRs and TPPS4	Nanometal surface energy transfer (NSET)	0.5 - 7.5	Human urine	0.04	-	2
Cu-PCE	Impedometric sensor	10^{-5} - 10^{-9} M	Urine and blood plasma	0.26	-	3
Cu2+ complex	Fluorescence and colorimetric sensor	0 – 20 & 0–2.6	Mushroom and meat Samples	3.62×10^{-2}	-	4
Ag-Au/AgCl nanohybrid	Colorimetric sensor	2.6×10^{-3} - 8.0	Urine sample	0.10	92–115 %	5
ssDNA-capped AuNPs	Colorimetric sensor	0.35–3.95	Urine & serum samples	6.87×10^{-2}	91.40 - 101.80%	6
Tyr-Au NPs	Colorimetric and fluorimetric sensor	1.00×10^{-7} to 0.05 & 0.01–0.13	Human plasma & urine samples	1.36×10^{-4} & 0.62×10^{-2}	99.30–101.60%	7

CuI-CPs	Colorimetric and fluorescent sensor	0–250	Meat Samples	0.57	-	8
**2D Azeno**	**Fluorescent sensor**	**0.005 – 450 µM**	**Human serum and urine**	**4.35×10^{-3} (0.88 ppb)**	**97-102 %**	**This Work**

Table S5. Comparison table showing a comparison of various chromatographic techniques and commercial techniques used for SPM detection and the developed 2D-Azeno HICTM sensor.

Method	Real sample	Linear Range	LOD (uM)	Recovery(%)	Ref.
HPLC-DAD (dansyl-Cl)	Cheese	24.7 - 988	7.6	70-120%	9
HPLC-UV (dansyl-Cl)	Aquatic products	0.49-247	0.069	68-123%	10
HPLC-APCI-MS (dansyl derivatives)	Baby food	0.0247-1.48	0.00826	86.0-105.2%	11
HPLC-UV (dansyl-Cl)	Chicken meat	14.3- 467	1.48	92.25-102.25%	12
UPLC-MS/MS (benzoyl-Cl)	Fish	0.002–8.0	0.00109	84.6-119.3%	13
HPLC-UV (dansyl-Cl)	Agricultural products	124-4942	0.049-0.49	84.85-103.34%	14
LC-ESI-MS/MS (SIFA derivatization)	Human plasma	0.01 – 1.0	0.0004	94-106%	15
HPTLC	Plant tissues	0.494–3.96	0.148	83.7–114.4%	16
HPLC-MS/MS	Whey samples	0.00366–49.4	0.00109	83.0-91.0%	17

Competitive ELISA (antigen-coated plate, HRP-TMB)	Human serum and plasma (spiked)	0.062-3.95	0.037	Serum (88-99%, (EDTA plasma)93-103%, (Heparin plasma)89-103%	[18]
Competitive ELISA (antigen-coated plate, HRP-TMB)	Human serum and plasma (spiked)	0.0025-0.050	0.0025	Not provided	[19]
Competitive ELISA (monoclonal anti-DiAcSpm)	Human urine	0.00625-0.20 (as DiAcSpm)	0.0125 (as DiAcSpm)	Not provided	[20]
Competitive ELISA (polyclonal anti-DiAcSpm)	Human urine (healthy + cancer patients)	0.003-0.050 (as DiAcSpm)	Not specified	80-115%	[21]
Competitive ELISA (polyclonal anti-DiAcSpm)	Human urine (breast + colorectal cancer patients)	Not individually specified	Not specified	Not provided	[22]
**2D-Azeno NSs (Fluorescence sensor)**	**Urine and Blood serum**	**0.005 – 450 µM**	**4.35×10^{-3}**	**97-102%**	**This Work**

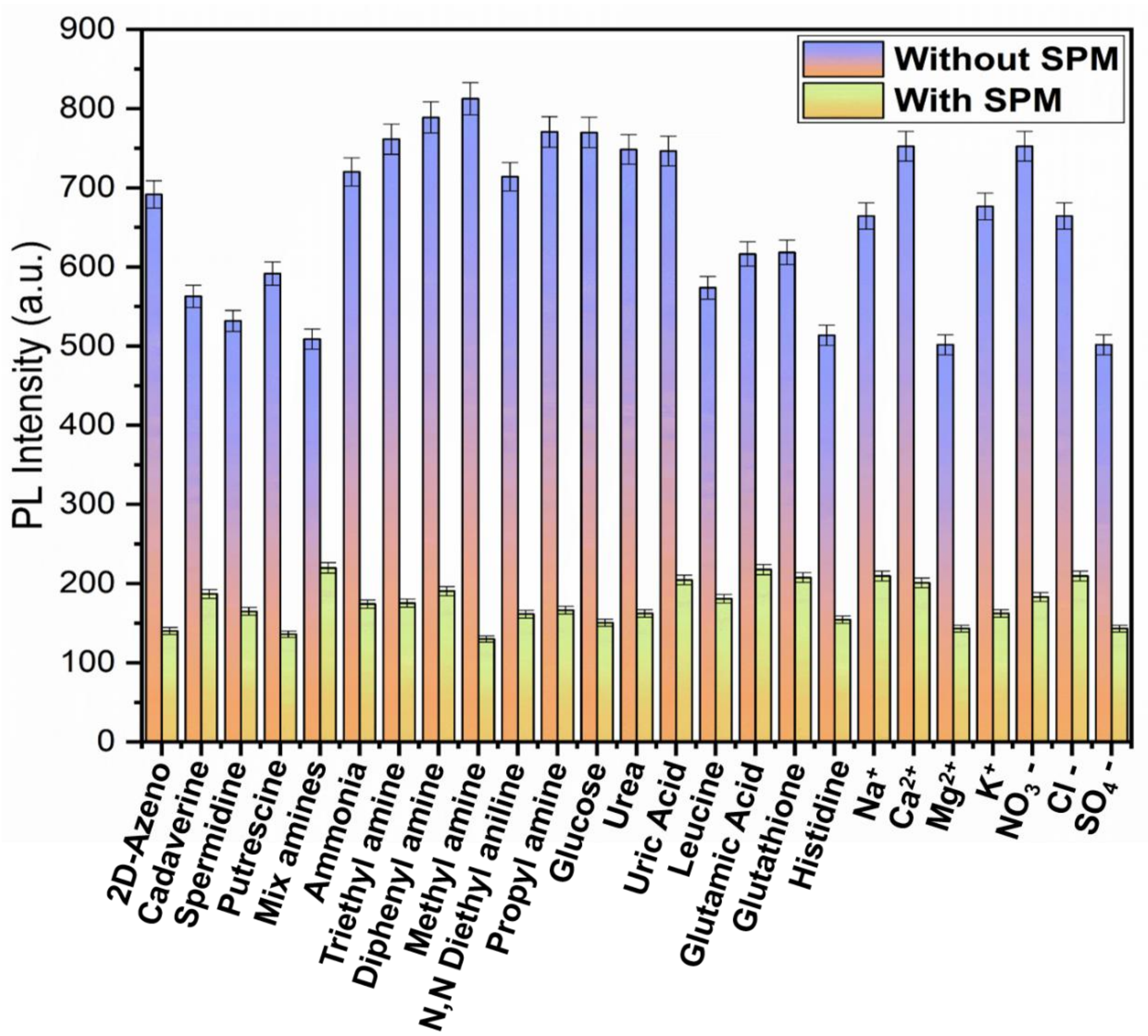

Figure S8. Anti-interference study for the detection of SPM in biological and food samples.

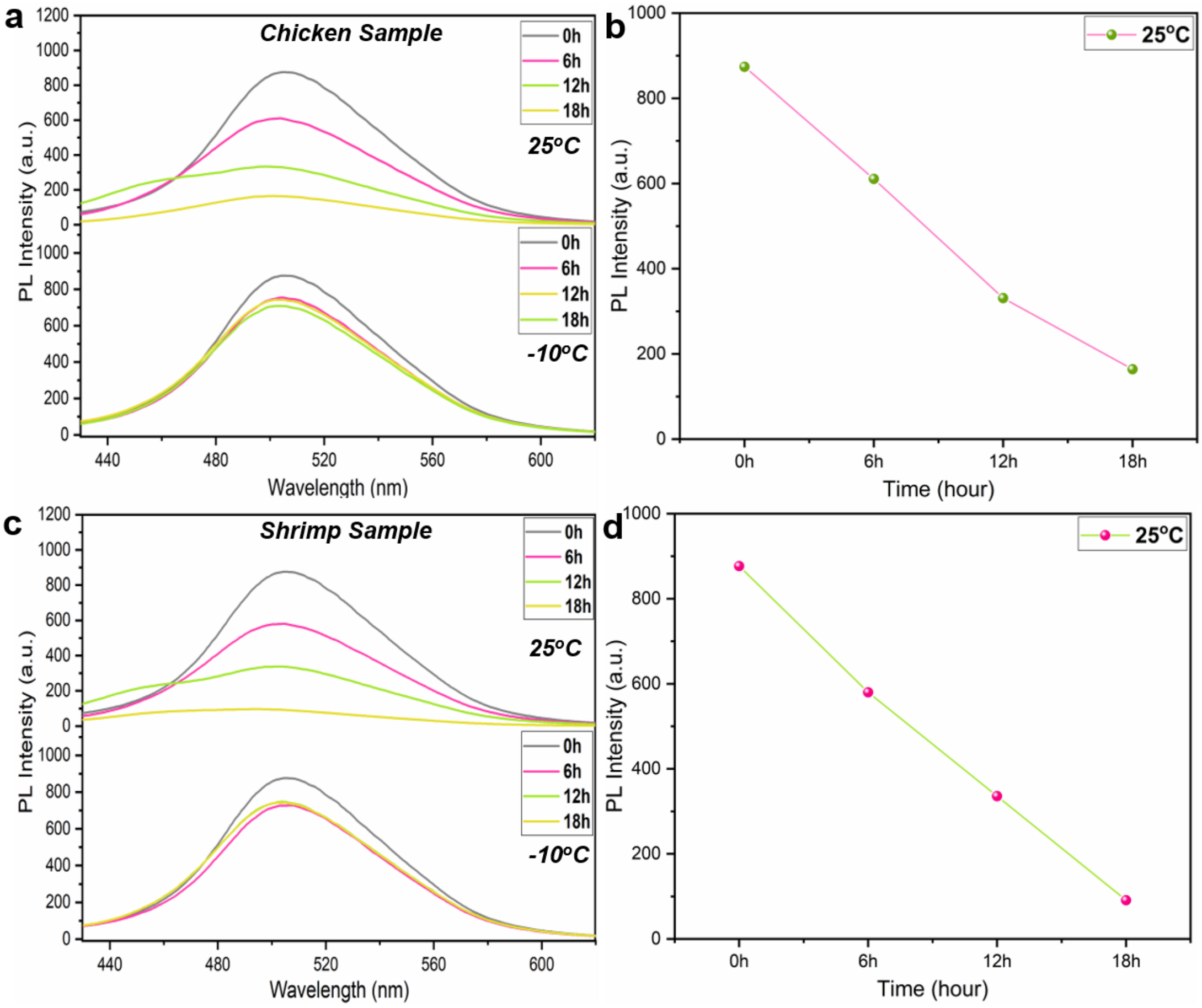

Figure S9. PL spectra showing fluorescence response of 2D-Azeno towards food samples stored at different temperatures (a,b) Chicken sample (c,d) Shrimp sample.

Table S6. Natural bond orbitals (NBO) analysis for 2D-Azeno NSs in gas phase and in DMSO.

**Atom**	**Charge (Gas phase)**	**Charge (DMSO)**	**ΔCharge (DMSO–Gas)**	**2p (Gas phase)**	**2p (DMSO)**	**Δ2p (DMSO–Gas)**	**Interpretation**
N21	-0.62718	-0.62179	-0.00539	4.31	4.32	0.01	Primary H-bond acceptor (most negative charge, high lone pair density)
N23	-0.62718	-0.62211	-0.00507	4.31	4.32	0.01	Primary H-bond acceptor (most negative charge, high lone pair density)
N27	-0.46947	-0.51147	-0.03273	4.11	4.15	0.04	Secondary H-bond acceptor (less negative; increased polarity in DMSO)
N28	-0.46947	-0.51176	-0.03282	4.11	4.15	0.04	Secondary H-bond acceptor (less negative; increased polarity in DMSO)
N29	-0.46948	-0.51143	-0.03271	4.11	4.15	0.04	Secondary H-bond acceptor (less negative; increased polarity in DMSO)
N30	-0.46948	-0.51141	-0.03276	4.11	4.15	0.04	Secondary H-bond acceptor (less negative;

							increased polarity in DMSO)

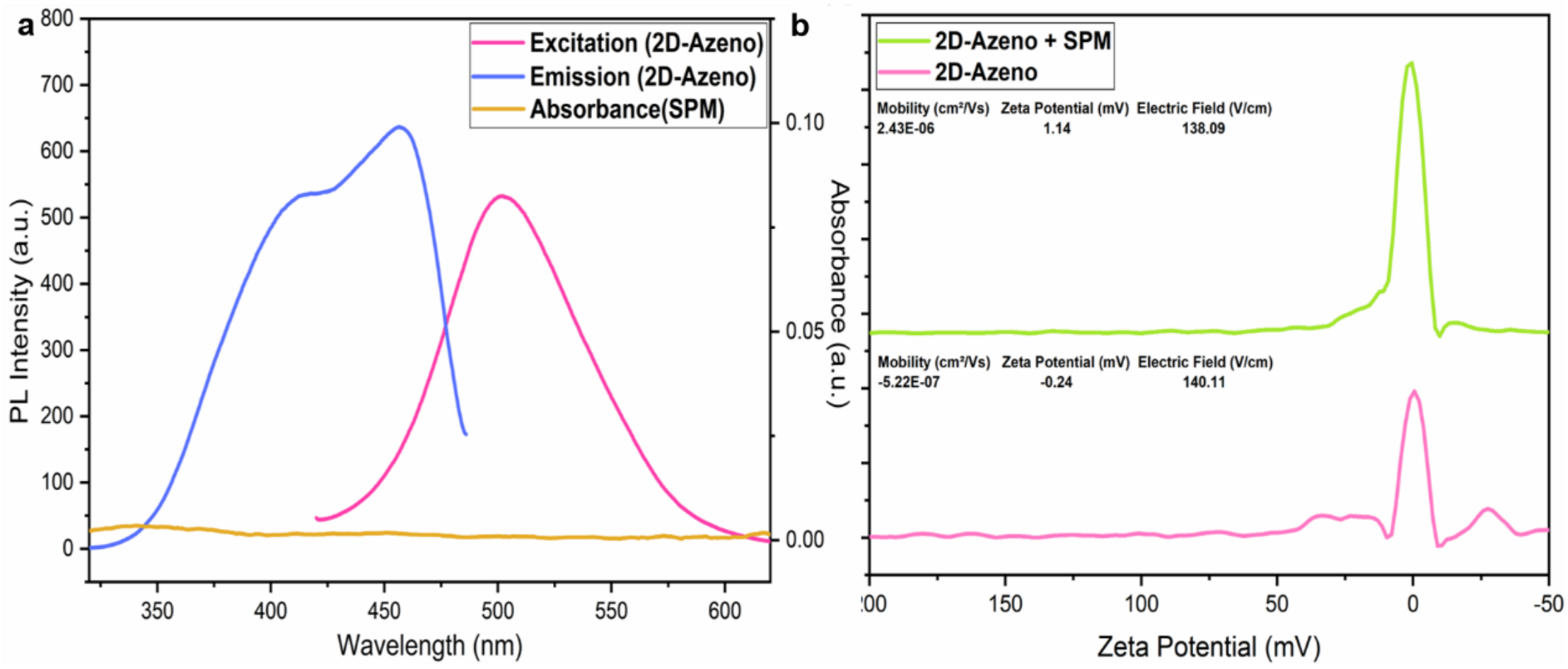

Figure S10. (a) Overlapping spectra for 2D-Azeno PL emission and excitation and SPM UV absorbance **(b)** Zeta potential of 2D-Azeno NSs before and after adding SPM.

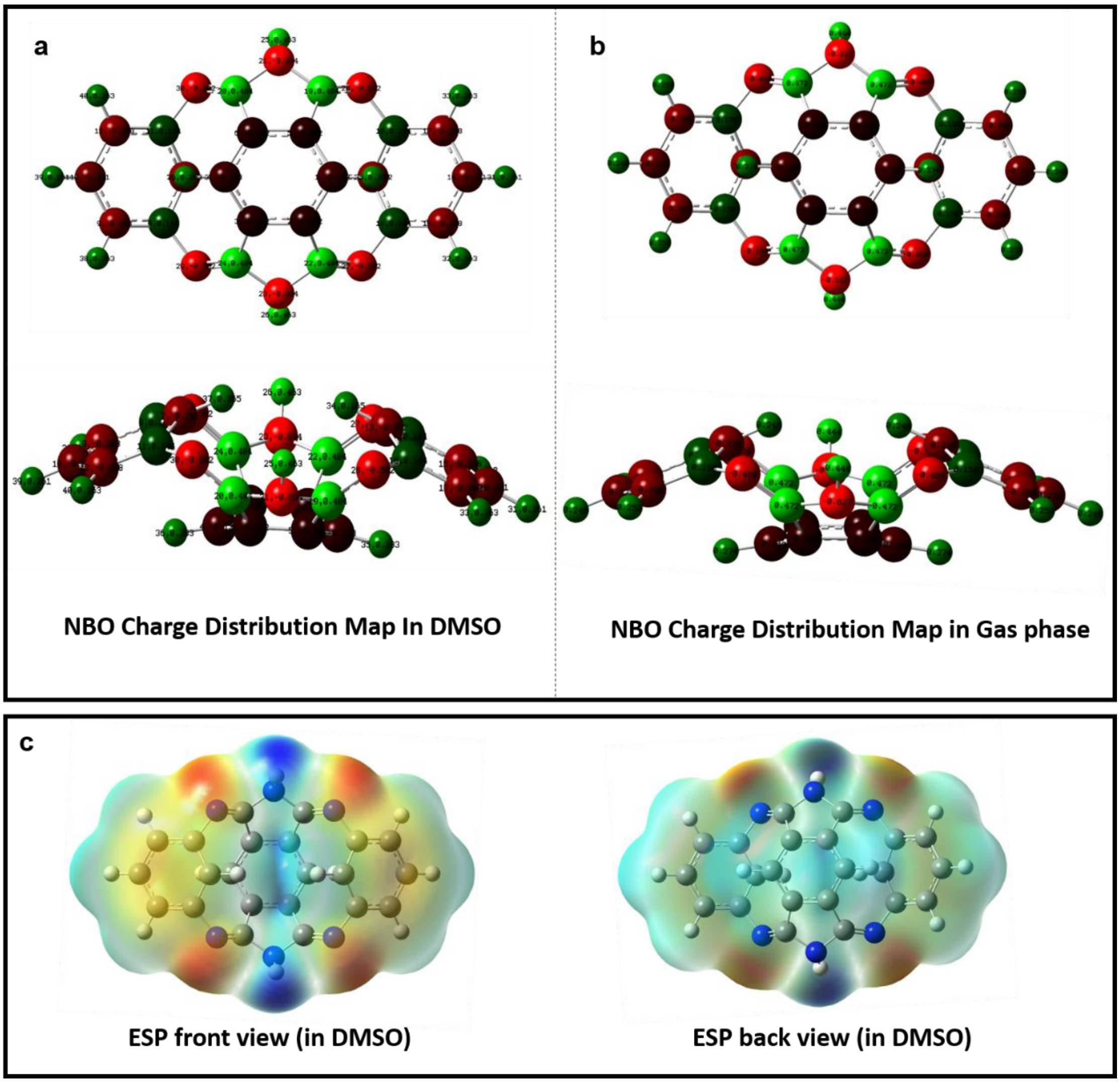

Figure S11. NBO charge distribution of 2D-Azeno NSs **(a)** in DMSO **(b)** in Gas phase **(c)** Electrostatic potential (ESP) map for 2D-Azeno NSs in DMSO.

Calculations:

1. <u>LOD Calculations:</u>

y = 0.4127x + 0.0219

Slope = 0.4127

$$\boldsymbol{LOD = 3.3 \times \frac{S.D}{Slope}}$$

SD = 0.544273

LOD = 3.3 × 0.544273/0.4127 = **4.35 nM**

2. <u>Quantum Yield Calculations:</u>

Following equation was used for quantum yield calculations:

$$\varphi_{2D-Azeno} = \varphi_{ref} \times \frac{F(AUC)_{2D-Azeno}}{F(AUC)_{ref}} \times \frac{Absorbance_{ref}}{Absorbance_{2D-Azeno}} \times \frac{(\eta_{2D-Azeno})^2}{(\eta_{ref})^2}$$

φ_{ref} : Quantum yield of Reference (Quinine Sulfate dihydrate) = 54.6 %

$\varphi_{2D-Azeno}$: Quantum yield of 2D-Azeno

F (AUC) $_{ref}$: Fluorescence Area under the curve of Reference

$F(AUC)_{2D-Azeno}$: Fluorescence Area under the curve of 2D-Azeno

$Absorbance_{ref}$: Absorbance of Reference

$Absorbance_{2D-Azeno}$: Absorbance of 2D-Azeno

η_{ref}: Solvent refractive index of the Reference: 1.333 (Water)

$\eta_{2D-Azeno}$: Solvent refractive index of the 2D-Azeno NSs: 1.477 (DMSO)

$$\varphi_{2D-Azeno} = 54.6\% \times \frac{67{,}825}{91{,}116} \times \frac{0.295}{0.427} \times \frac{2.18}{1.78}$$

$\boldsymbol{\varphi_{2D-Azeno} = 34.19\%}$

References

(1) Luhar, S.; Ghosh, R.; Chatterjee, P. B.; Srivastava, D. N.An Impedometric Sensor Based on Boronic Acid @ Plastic Chip Electrode for Sensitive Detection of Prostate Cancer Biomarker Spermine. *Biosensors and Bioelectronics: X* **2022**, *12*, 100219. https://doi.org/https://doi.org/10.1016/j.biosx.2022.100219.

(2) Yuan, D.; Liu, J. J.; Zhang, H. Z.; Wang, N.; Zou, H. Y.; Huang, C. Z.; Wang, J.Highly Selective Detection of Spermine in Human Urine via a Nanometal Surface Energy Transfer Platform. *Talanta* **2018**, *188*, 218–224. https://doi.org/https://doi.org/10.1016/j.talanta.2018.05.086.

(3) Ghosh, R.; Luhar, S.; Debnath, S.; Patel, K. B.; Baskaran, K.V; Srivastava, D. N.; Chatterjee, P. B.Measurement of the Malignancy Marker Spermine in Human Biofluids Using Smartphone Readout and Impedance Techniques: Analytical Validation Using HPLC. *Sensors and Actuators B: Chemical* **2024**, *406*, 135390. https://doi.org/https://doi.org/10.1016/j.snb.2024.135390.

(4) Chopra, S.; Singh, A.; Venugopalan, P.; Singh, N.; Kaur, N.Organic Nanoparticles for Visual Detection of Spermidine and Spermine in Vapors and Aqueous Phase. *ACS Sustainable Chemistry & Engineering* **2017**, *5* (2), 1287–1296. https://doi.org/10.1021/acssuschemeng.6b01295.

(5) Kuo, P.-C.; Lien, C.-W.; Mao, J.-Y.; Unnikrishnan, B.; Chang, H.-T.; Lin, H.-J.; Huang, C.-C.Detection of Urinary Spermine by Using Silver-Gold/Silver Chloride Nanozymes. *Analytica Chimica Acta* **2018**, *1009*, 89–97. https://doi.org/https://doi.org/10.1016/j.aca.2018.01.018.

(6) Liu, Z.De; Zhu, H. Y.; Zhao, H. X.; Huang, C. Z.Highly Selective Colorimetric Detection of Spermine in Biosamples on Basis of the Non-Crosslinking Aggregation of SsDNA-Capped Gold Nanoparticles. *Talanta* **2013**, *106*, 255–260. https://doi.org/https://doi.org/10.1016/j.talanta.2012.10.079.

(7) Rawat, K. A.; Bhamore, J. R.; Singhal, R. K.; Kailasa, S. K.Microwave Assisted Synthesis of Tyrosine Protected Gold Nanoparticles for Dual (Colorimetric and Fluorimetric) Detection of Spermine and Spermidine in Biological Samples. *Biosensors and Bioelectronics* **2017**, *88*, 71–77. https://doi.org/https://doi.org/10.1016/j.bios.2016.07.069.

(8) Shi, Z.; Wu, Y.; Zhang, X.; Gao, Y.; Qiao, L.; Hou, Q.; Wang, M.Highly Sensitive and Selective Colorimetric and Fluorescent Dual-Readout Assay for the Analysis of Spermine, Spermidine and Cysteine. *Dyes and Pigments* **2023**, *220*, 111659. https://doi.org/https://doi.org/10.1016/j.dyepig.2023.111659.

(9) Pawul-gruba, M.; Kiljanek, T.; Madejska, A.; Osek, J.Development of a High Performance Liquid Chromatography with Diode Array Detector (HPLC-

DAD) Method for Determination of Biogenic Amines in Ripened Cheeses. **2022**.
(10) Fu, Q.; Zheng, H.; Han, X.; Cao, L.; Sui, J.Development of a Highly Sensitive HPLC Method for the Simultaneous Determination of Eight Biogenic Amines in Aquatic Products. *Acta Chromatographica* **2021**, *33* (4), 378–386. https://doi.org/10.1556/1326.2020.00824.
(11) Czajkowska-Mysłek, A.; Leszczyńska, J.Liquid Chromatography–Single-Quadrupole Mass Spectrometry as a Responsive Tool for Determination of Biogenic Amines in Ready-to-Eat Baby Foods. *Chromatographia* **2018**, *81* (6), 901–910. https://doi.org/10.1007/s10337-018-3527-z.
(12) Assis, D. C. S.; Menezes, L. D. M.; Lima, A. L.; Klein, R. W. T.; Heneine, L. G. D.; Lara, L. J. C.; Teixeira, L.V.; Ornellas, C. B. D.; Cançado, S.V.; Figueiredo, T. C.Validation of an HPLC-UV Method for the Identification and Quantification of Bioactive Amines in Chicken Meat. *Arquivo Brasileiro de Medicina Veterinaria e Zootecnia* **2016**, *68* (3), 805–813. https://doi.org/10.1590/1678-4162-8668.
(13) Amines, B.; Wang, R.; Wang, P.The Development of an Ultra-Performance Liquid Chromatography – Tandem Mass Spectrometry Method For. **2023**.
(14) Yoon, H.; Park, J. H.; Choi, A.; Hwang, H. J.; Mah, J. H.Validation of an HPLC Analytical Method for Determination of Biogenic Amines in Agricultural Products and Monitoring of Biogenic Amines in Korean Fermented Agricultural Products. *Toxicological Research* **2015**, *31* (3), 299–305. https://doi.org/10.5487/TR.2015.31.3.299.
(15) Deng, Y. H.; Zhang, H. S.; Du, X. L.; Wang, H.Quantification of Biogenic Amines in Human Plasma Based on the Derivatization with N-Hydroxy-Succinimidyl Fluorescein-O-Acetate by High-Performance Liquid Chromatography. *Journal of Separation Science* **2008**, *31* (6–7), 990–998. https://doi.org/10.1002/jssc.200700399.
(16) Mohajeri, M.; Ayatollahi, S. A.; Khandan, M.; Mokhtari, S.; Hosseinzadeh, V.; Kobarfard, F.A Simple, Easy, and Efficient HPTLC Method for Simultaneous Determination of Polyamines (Putrescine, Spermidine, and Spermine) in Plant Tissues. *Journal of Food Composition and Analysis* **2024**, *126* (August 2023), 105835. https://doi.org/10.1016/j.jfca.2023.105835.
(17) Mirzaei, H.; Afshar Mogaddam, M. R.; Khandaghi, J.Simultaneous Determination of Four Biogenic Amines in Whey Samples Using a New Solid Phase Extraction Method Prior to Their Analysis by HPLC-MS/MS. *Microchemical Journal* **2022**, *177* (February), 107313. https://doi.org/10.1016/j.microc.2022.107313.

(18) Kit, S. E.Spermine ELISA Kit. 2–8.
(19) Kit, S. E.Spermine ELISA Kit. **2025**, *49* (0), 1–7.
(20) Company, K. B.Urinary Diacetylspermine ELISA. **1971**, 1–5.
(21) Masao, M.Two Enzyme-Linked - Diacetylspermidine Antibodies Immunosorbent Assay (ELISA) Systems for N1 , N8 Using Monoclonal. **2002**, *132* (5).
(22) Umemori, Y.; Ohe, Y.; Kuribayashi, K.; Tsuji, N.; Nishidate, T.; Kameshima, H.; Hirata, K.; Watanabe, N.Evaluating the Utility of N1,N12-Diacetylspermine and N1,N8-Diacetylspermidine in Urine as Tumor Markers for Breast and Colorectal Cancers. *Clinica Chimica Acta* **2010**, *411* (23–24), 1894–1899. https://doi.org/10.1016/j.cca.2010.07.018.
